# Photophysical Integrity of the Iron(III) Scorpionate Framework in Iron(III)–NHC Complexes with Long-Lived ^2^LMCT Excited States

**DOI:** 10.1021/acs.inorgchem.2c02410

**Published:** 2022-10-24

**Authors:** Om Prakash, Linnea Lindh, Nidhi Kaul, Nils W. Rosemann, Iria Bolaño Losada, Catherine Johnson, Pavel Chábera, Aleksandra Ilic, Jesper Schwarz, Arvind Kumar Gupta, Jens Uhlig, Tore Ericsson, Lennart Häggström, Ping Huang, Jesper Bendix, Daniel Strand, Arkady Yartsev, Reiner Lomoth, Petter Persson, Kenneth Wärnmark

**Affiliations:** † Centre for Analysis and Synthesis, Department of Chemistry, Lund University, Box 124, SE-22100Lund, Sweden; ‡ Chemical Physics Division, Department of Chemistry, Lund University, Box 124, SE-22100Lund, Sweden; § Theoretical Chemistry Division, Department of Chemistry, Lund University, Box 124, SE-22100Lund, Sweden; ∥ Department of Chemistry − Ångström Laboratory, Uppsala University, Box 523, SE-75120Uppsala, Sweden; ⊥ Department of Physics − Ångström Laboratory, Uppsala University, Box 523, SE-75120Uppsala, Sweden; # Department of Chemistry, University of Copenhagen, Universitetsparken 5, DK-2100Copenhagen, Denmark

## Abstract

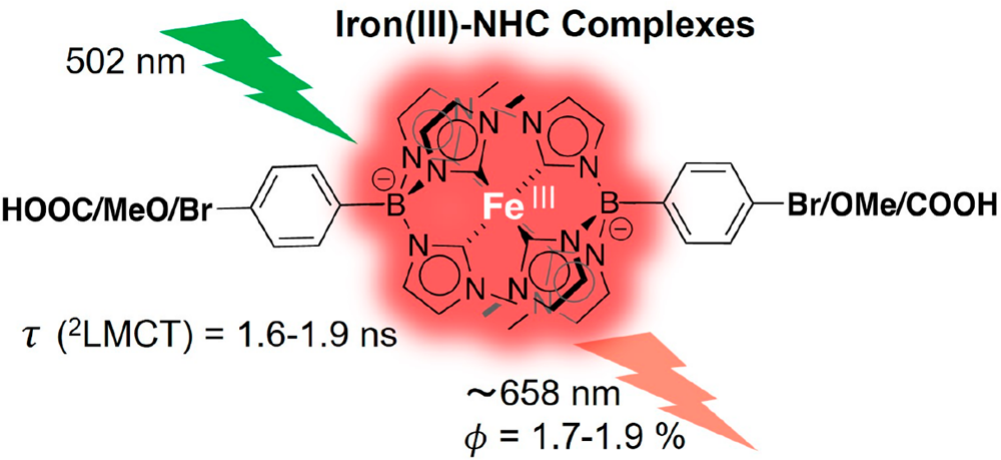

Fe(III) complexes with *N*-heterocyclic carbene (NHC) ligands belong to the rare examples of Earth-abundant transition metal complexes with long-lived luminescent charge-transfer excited states that enable applications as photosensitizers for charge separation reactions. We report three new *hexa*-NHC complexes of this class: [Fe(brphtmeimb)_2_]PF_6_ (brphtmeimb = [(4-bromophenyl)tris(3-methylimidazol-2-ylidene)borate]^–^, [Fe(meophtmeimb)_2_]PF_6_ (meophtmeimb = [(4-methoxyphenyl)tris(3-methylimidazol-2-ylidene)borate]^–^, and [Fe(coohphtmeimb)_2_]PF_6_ (coohphtmeimb = [(4-carboxyphenyl)tris(3-methylimidazol-2-ylidene)borate]^–^. These were derived from the parent complex [Fe(phtmeimb)_2_]PF_6_ (phtmeimb = [phenyltris(3-methylimidazol-2-ylidene)borate]^–^ by modification with electron-withdrawing and electron-donating substituents, respectively, at the 4-phenyl position of the ligand framework. All three Fe(III) *hexa*-NHC complexes were characterized by NMR spectroscopy, high-resolution mass spectroscopy, elemental analysis, single crystal X-ray diffraction analysis, electrochemistry, Mößbauer spectroscopy, electronic spectroscopy, magnetic susceptibility measurements, and quantum chemical calculations. Their ligand-to-metal charge-transfer (^2^LMCT) excited states feature nanosecond lifetimes (1.6–1.7 ns) and sizable emission quantum yields (1.7–1.9%) through spin-allowed transition to the doublet ground state (^2^GS), completely in line with the parent complex [Fe(phtmeimb)_2_]PF_6_ (2.0 ns and 2.1%). The integrity of the favorable excited state characteristics upon substitution of the ligand framework demonstrates the robustness of the scorpionate motif that tolerates modifications in the 4-phenyl position for applications such as the attachment in molecular or hybrid assemblies.

## Abstract 2

A series of well-defined Fe(III) complexes through a hexa-NHC scorpionate framework with electron-donating and -withdrawing substituents were synthesized and fully characterized. All three Fe(III) complexes show the visible photoluminescence around 630 nm at room temperature through ligand-to-metal charge-transfer (^2^LMCT) excited states with nanosecond lifetimes (1.6−1.7 ns) and generous emission quantum yields (1.7−1.9%) through spin-allowed transition to the doublet ground state (2GS), exclusively similar to the parent complex [Fe(phtmeimb)_2_]PF_6_ (2.0 ns and 2.1%).

## Introduction

The development of photosensitizers based on Earth-abundant, inexpensive, and nontoxic metals, with the goal of replacing the to-date widely used noble metals, has attracted a lot of interest in the field of coordination chemistry in recent years.^1^ Such research is motivated by the desire to use solar-energy conversion processes on a large scale. Until recently, the field of solar energy conversion based on coordination compounds has to a large degree focused on octahedral metal complexes of noble metals with low-spin 4d^6^ or 5d^6^ electronic configurations using different second and third row transition metals (TMs) including Ru(II), Re(I), Os(II), and Ir(III).^2,3^ The ligand field splitting in such transition metal complexes is inherently larger than that for the corresponding complexes containing first row TMs such as Cr, Mn, Fe, and Co. This shifts the metal-centered (MC) states to higher energies in the former case, which in turn results in slow deactivation of the photoactive charge-transfer (CT) states.^4^ Together with the employment of π-accepting ligands such as 2,2-bipyridyl (bpy), in metal complexes involving 4d^6^ or 5d^6^ metal cations, this has led to the development of many metal complexes that have metal-to-ligand charge-transfer (MLCT) states lower than MC states in energy.^5,6^ This implies that, for the MLCT state, a state with demonstrated importance for photofunctional applications for metal complexes based on 4d^6^ and 5d^6^ metal cations, the deactivation of the excited state via the MC states is a concern only at elevated temperatures.^5^ Efficient intersystem crossing from ^1^MLCT states usually populates ^3^MLCT states that exhibit slow radiative and nonradiative relaxation to the ground state. Additionally, 4d^6^ and 5d^6^ metal complexes with π accepting ligands display a relatively wide visible light absorption window and favorable redox properties of the GS and the ^3^MLCT state. As a result, they are heavily featured in photophysical applications.^2^ In parallel, four-coordinate 5d^8^ complexes involving Pt(II) and Au(III) have also been investigated and successfully used in photophysical applications thanks to the strong ligand field connected to these third-row transition metals.^5,7^

There have been some reports about photoactive Earth-abundant metal complexes, foremost from metal complexes containing TMs such as Cu(I), Cr(0), Mn(I/IV), and Co(III).^5^ The problem with first row transition metal complexes for photophysical applications in general is that the weak ligand field results in their MC states being relatively low in energy, providing a fast deactivation pathway and reducing the efficiency of the photofunctional MLCT states.^4^ Of the first row transition metals, iron is by far the most abundant.^8^ For Fe(II) polypyridyl complexes, the most widely studied direct base metal analogues of the successful Ru-, Os- and Ir-polypyridyl photosensitizers, the MLCT states are deactivated on the 100 fs time scale to the low-lying MC states.^6^ McCusker and Heinze reported attempts to increase the MLCT excited state lifetime by employing ligands with increased bite angle and introducing π-accepting and/or push–pull moieties.^9,10^ Recently, McCusker reported a cage compound involving the Fe(II)(bpy)_3_ motif, exhibiting a 2.6 ps MLCT lifetime, the longest recorded to date for an iron polypyridine complex.^11^ However, the introduction of strongly σ-donating *N*-heterocyclic carbene (NHC) ligands in the field of photoactive iron complexes^6,12^ has significantly increased the excited state lifetime of Fe(II) MLCT states, reaching up to 528 ps.^13^ By increasing the ligand field strength, the MC states increase in energy, thus slowing down the deactivation of the MLCT state.^6,14,15^ The photophysical properties of Fe–NHC metal complexes have been further improved using different approaches.^16−20^

By employing the facial tridentate scorpionate pre-NHC ligand [phtmeimbH_3_](PF_6_)_2_ (where phtmeimbH_3_ = [phenyltris(3-methyl-1*H*-imidazol-3-ium-1-yl)borate)]^2+^), reported by Smith,^21^ based on the corresponding pre-NHC ligand originally developed by Fehlhammer,^22^ [htmeimbH_3_](PF_6_)_2_ (where htmeimbH_3_ = [hydridotris(3-methyl-1*H*-imidazol-3-ium-1-yl)borate)]^2+^), we synthesized the corresponding Fe(III) complex [Fe(phtmeimb)_2_]PF_6_ (where phtmeimb = [phenyltris(3-methylimidazol-2-ylidene)borate]^–^).^23^ This complex showed an LMCT excited-state lifetime of 2 ns and an intense fluorescence with a 2.1% quantum yield,^23^ constituting the second example of room temperature photoluminescence from an iron complex.^24^ Further, the LMCT excited state was oxidatively and reductively quenched in bimolecular reactions using standard electron donors and acceptors, which was the first example of such quenching involving an iron charge-transfer state being demonstrated.^23,25^ Very recently, Therien synthesized an Fe–NHC–porphyrin conjugate that showed photoluminescence from a state with considerable MLCT contribution,^26^ and Bauer found both MLCT and LMCT photoluminescence from photoexcited states of an Fe(III)–NHC-cyclometalated complex, as communicated in a preliminary report.^27^ In fact, there are only three examples of iron complexes with a nanosecond excited CT state lifetime outside the class of NHC complexes, namely, the Fe(II) complexes reported by Herbert involving strongly electron-donating amide ligands^28^ and the cyclometalated Fe(II) complex with a phenylphenanthroline framework reported by Berkefeld.^29^

Given the few examples of complexes with iron-based photoluminescence and/or long-lived iron-based CT states, it is clearly a challenging task to generate new iron-based complexes possessing photophysical properties that allow for efficient applications. However, there is an interest in modifying existing, promising iron NHC complexes, as described by the increase in reported Fe–NHC complexes from 2013 to date.^14,15^ Most new complexes are based on structural variations of the ligand framework of the first iron complex having a ^3^MLCT lifetime above 1 ps, the Fe–NHC complex [Fe(pbmi)_2_](PF_6_)_2_ (where pbmi = 1,1′-(pyridine-2,6-diyl)bis(3-methylimidazol-2-ylidene)).^12^

**Figure 1:**
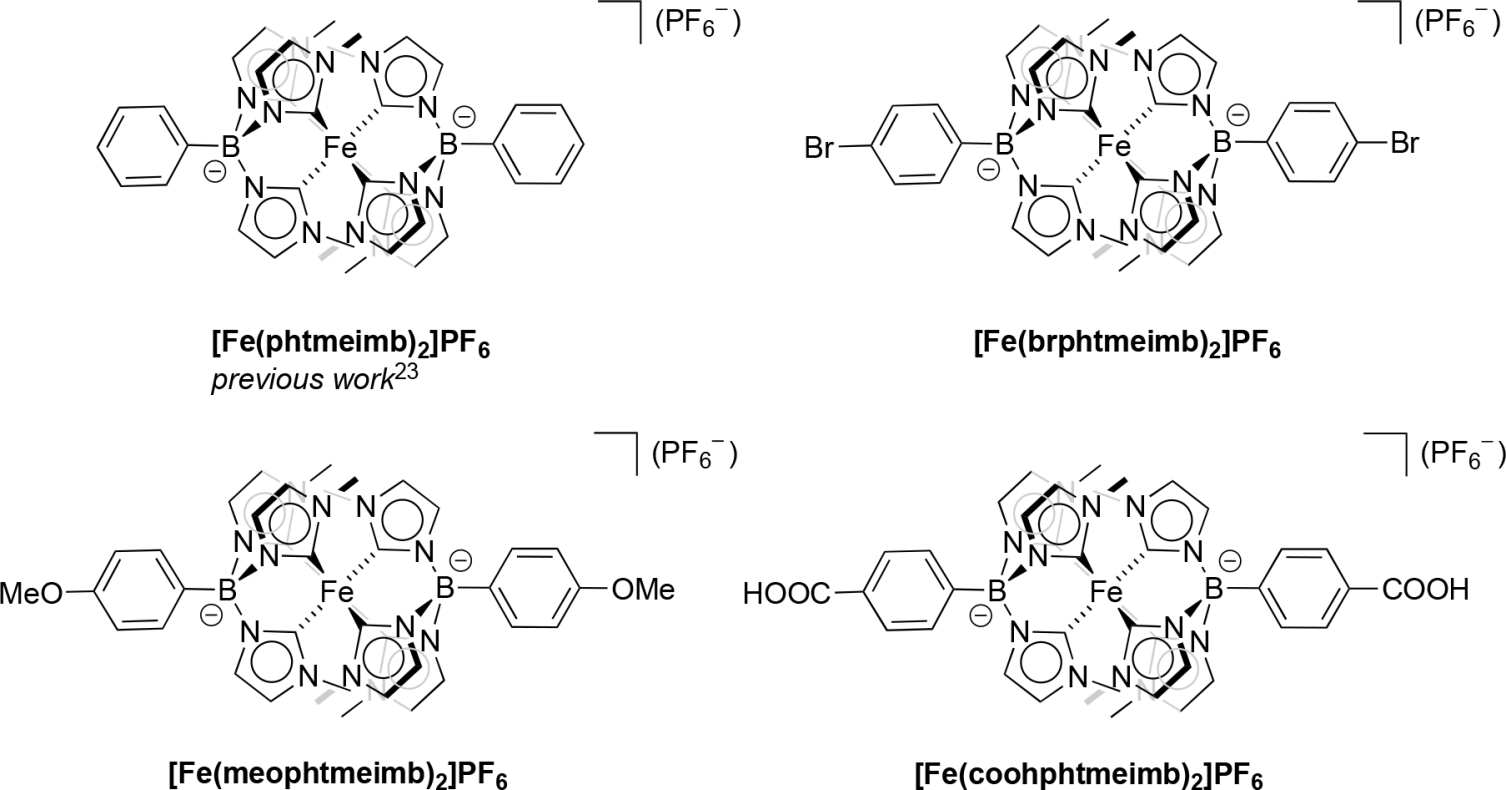
Chemical structures of the Fe(III) complexes investigated in this study.

Here, we report the first series of modifications of [Fe(phtmeimb)_2_]PF_6_ with the purpose of finding possible structure–(photo)functional relationships based on the presence of electron-withdrawing and -donating substituents. To this end, the 4-position of the phenyl group present in the framework of the NHC scorpionate ligand [phtmeimbH_3_]^−^ was substituted with either bromo, carboxyl, or methoxy groups, respectively. The resulting Fe(III) complexes [Fe(brphtmeimb)_2_]PF_6_, [Fe(meophtmeimb)_2_]PF_6_, and [Fe(coohphtmeimb)_2_]PF_6_ are shown in Figure 1. The 4-phenyl position was chosen, as it constitutes an obvious point of further functionalization when considering possibilities for application, for instance, for immobilization of the [Fe(phtmeimb)_2_]PF_6_ framework on a surface, allowing for potential photofunctional applications based on the [Fe(phtmeimb)_2_]^+^ chromophore attached to semiconductors, such as dye-sensitized solar cells^30,31^ and photocatalysts.^32,33^

## Results and Discussion

### Synthesis and Spectroscopic Characterizations

**Scheme 1:**
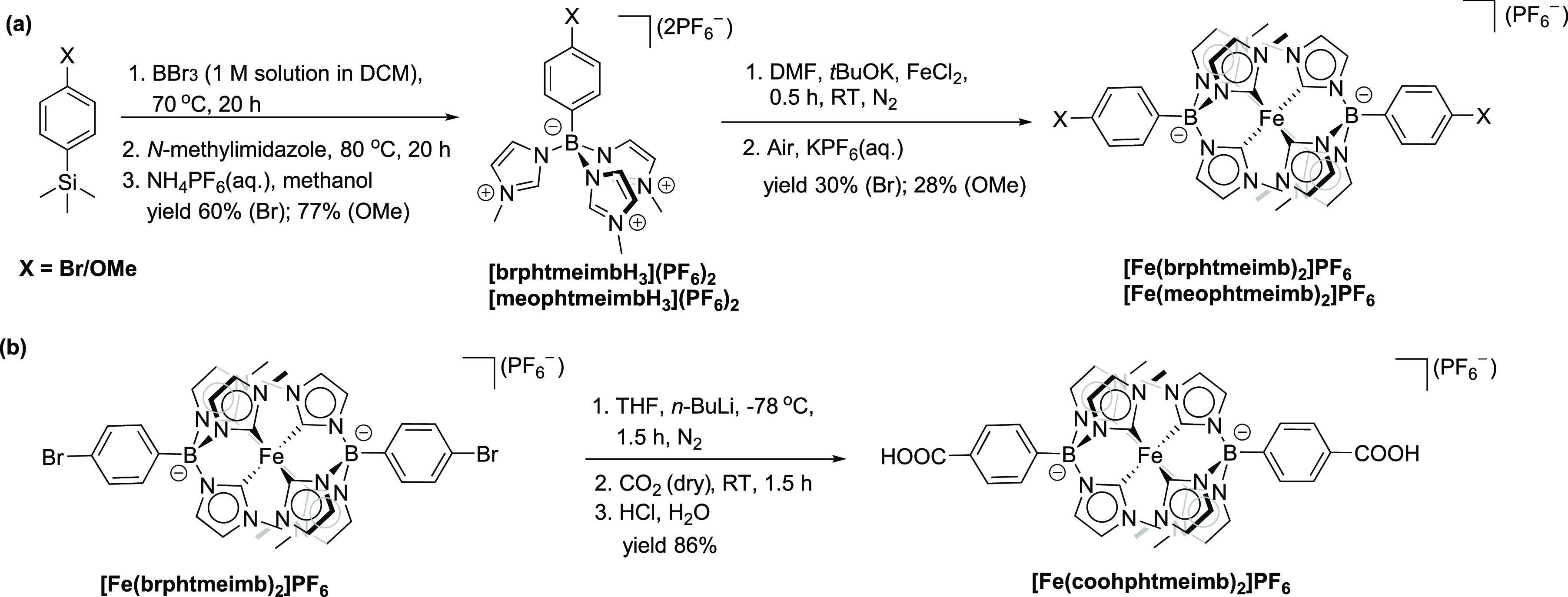
(a) Synthesis of [brphtmeimbH_3_](PF_6_)_2_, [Fe(brphtmeimb)_2_]PF_6_, [meophtmeimbH_3_](PF_6_)_2_, and [Fe(meophtmeimb)_2_]PF_6_; (b) Synthesis of [Fe(coohphtmeimb)_2_]PF_6_

The tridentate facial pre-NHC ligands [brphtmeimbH_3_](PF_6_)_2_ (brphtmeimbH_3_ = [(4-bromophenyl)tris(3-methyl-1*H*-imidazol-3-ium-1-yl)borate]^2+^ and [meophtmeimbH_3_](PF_6_)_2_ (meophtmeimbH_3_ = [(4-methoxyphenyl)tris(3-methyl-1*H*-imidazol-3-ium-1-yl)borate]^2+^ were synthesized as shown in Scheme 1a, following the synthetic procedure of the parent pre-NHC ligand [phtmeimbH_3_](PF_6_)_2_,^23^ starting from commercially available 4-bromophenyltrimethylsilane and 4-methoxyphenyltrimethylsilane, respectively. The iron(III) complexes [Fe(brphtmeimb)_2_]PF_6_ and [Fe(meophtmeimb)_2_]PF_6_ were efficiently synthesized under a nitrogen atmosphere from Fe^II^Cl_2_ and the corresponding free carbene ligands, generated *in situ* from [brphtmeimbH_3_](PF_6_)_2_ and [meophtmeimbH_3_](PF_6_)_2_, respectively, followed by spontaneous oxidation of Fe(II) into Fe(III) during the workup procedure in air, as shown in Scheme 1a (for details, see Supporting Information section S1). The iron(III) complex [Fe(coohphtmeimb)_2_]PF_6_ (coohphtmeimb = [(4-carboxyphenyl)tris(3-methyl-1*H*-imidazol-3-ium-1- yl)borate]^2+^ was synthesized by carboxylation of [Fe(brphtmeimb)_2_]PF_6_ using *n*-BuLi and CO_2_, as shown in Scheme 1b (for details, see Supporting Information section S1). The identity and purity of [Fe(brphtmeimb)_2_]PF_6_, [Fe(meophtmeimb)_2_]PF_6_, and [Fe(coohphtmeimb)_2_]PF_6_ were established by ^1^H NMR and ^13^C NMR spectroscopy, high-resolution mass spectrometry, elemental analysis, single crystal X-ray diffraction (scXRD), cyclic voltammetry, ^57^Fe Mößbauer spectroscopy, magnetic susceptibility measurements, and electron paramagnetic resonance spectroscopy.

**Figure 2:**
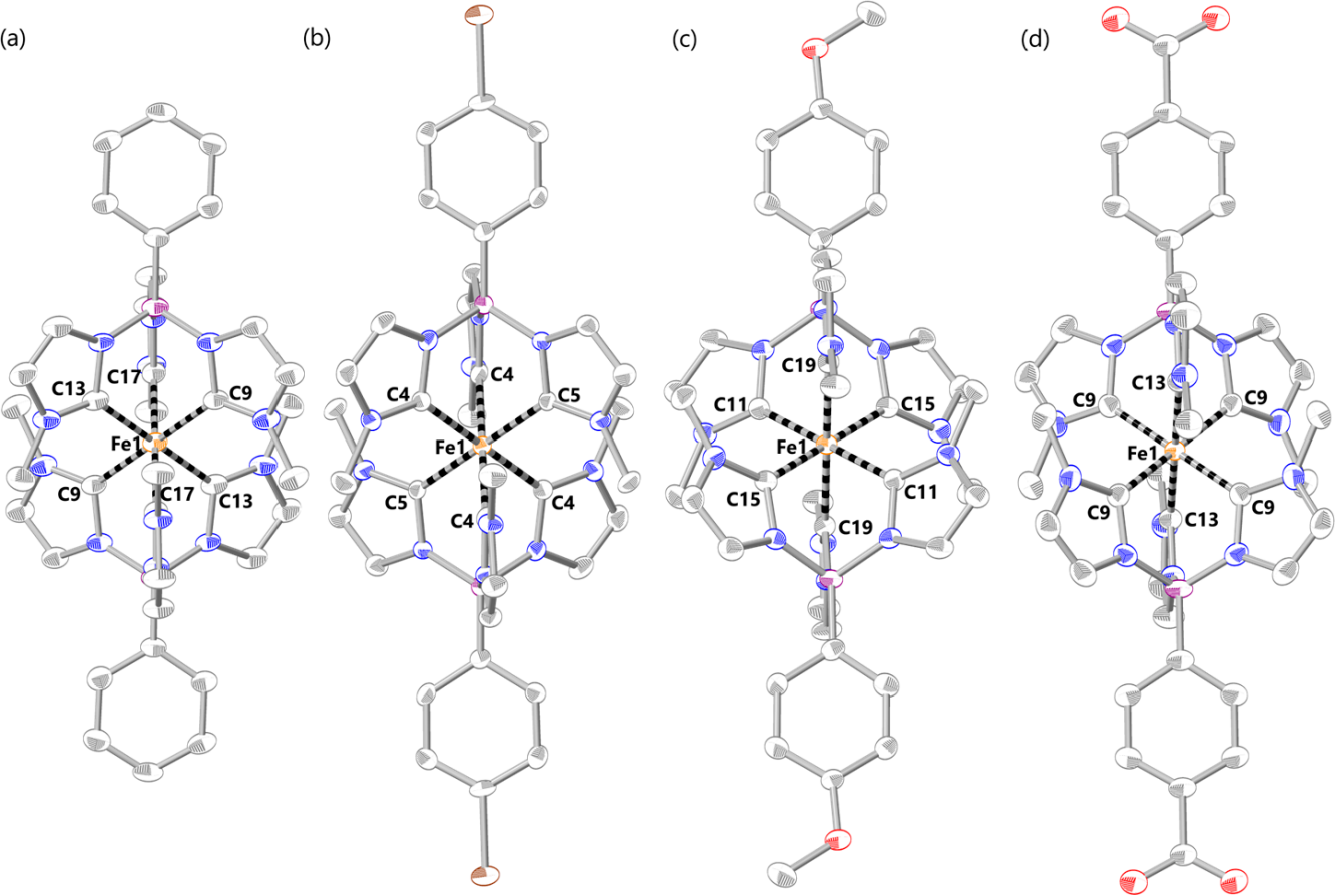
scXRD Molecular structures of [Fe(phtmeimb)_2_]PF_6_^23^ (a), [Fe(brphtmeimb)_2_]PF_6_ (b), [Fe(meophtmeimb)_2_]PF_6_ (c), and [Fe(coohphtmeimb)_2_]PF_6_ (d). Thermal ellipsoids are shown at 50% probability. Hydrogen atoms, counterions, and solvent molecules are omitted for clarity. Fe = orange; B = purple; N = blue; C = gray; Br = brown; O = red.

The ^1^H NMR and ^13^C{^1^H} NMR spectra of pre-NHC ligands [brphtmeimbH_3_](PF_6_)_2_ and [meophtmeimbH_3_](PF_6_)_2_ together with the metal complexes [Fe(brphtmeimb)_2_]PF_6_, [Fe(meophtmeimb)_2_]PF_6_, and [Fe(coohphtmeimb)_2_]PF_6_ coincided well with their proposed chemical structures. All metal complexes showed well-resolved ^1^H NMR signals in the range from 15 to −13 ppm, despite being d^5^ complexes (for details, see Supporting Information section S2), as previously observed for the parent complex [Fe(phtmeimb)_2_]PF_6_.^23^ Dark red single crystals of [Fe(brphtmeimb)_2_]PF_6_ and [Fe(meophtmeimb)_2_]PF_6_ suitable for scXRD analysis were grown from a saturated acetonitrile solution of the respective complex by slow diffusion of diethyl ether at room temperature. Purple single crystals of [Fe(coohphtmeimb)_2_]PF_6_ suitable for scXRD analysis were grown from a saturated acetonitrile:methanol (3:2) solution of the complex by slow diffusion of diethyl ether at room temperature (see Supporting Information section S4 for details). The molecular structure (Figure 2) and the +III oxidation state of iron in all complexes were unambiguously confirmed by scXRD analysis. The coordination of the iron center to the *bis*-tridentate NHC ligand resulted in the complexes [Fe(brphtmeimb)_2_]PF_6_, [Fe(meophtmeimb)_2_]PF_6_, and [Fe(coohphtmeimb)_2_]PF_6_, which resulted in a near-perfect octahedral geometry. The average Fe–C bond lengths and C–Fe–C bite angles are 2.008–1.976 Å and 87.28–86.70°, respectively, in all complexes. The observed bond lengths and angles are very similar (1.996 Å and 86.9°, respectively) to the reported parent [Fe(phtmeimb)_2_]PF_6_ complex (for details, see Supporting Information section S5).^23^ Notably, there are no significant deviations in the observed Fe–C bond length and C–Fe–C angles between the complexes substituted with −Br, −OMe, and −COOH groups at the 4-phenyl position in the respective ligand.

### Magnetism, EPR, and Mößbauer Study

The magnetization study for [Fe(brphtmeimb)_2_]PF_6_, [Fe(meophtmeimb)_2_]PF_6_, and [Fe(coohphtmeimb)_2_]PF_6_ is shown in Supporting Information Figure S14, together with data for the parent [Fe(phtmeimb)_2_]PF_6_ complex. The magnetization (*M*) for all three systems shows the expected variation with the reduced field (BT^–1^) indicating a saturation not much above 1 Bohr magneton (*μ*_*β*_). This agrees with the common *S* = ^1^/_2_ ground state for the expected low-spin electronic configuration (t_2g_^5^) imposed by the strong-field carbene ligands. For all three systems, the isothermally measured magnetization curves are superimposable in the (*BT*^–1^) plot, demonstrating the absence of zero field splitting as required for the low-spin (LS) t_2g_^5^ ground state. The temperature variations of the magnetic susceptibilities of the three systems are slightly different. In all three cases, the χ*T* product shows a temperature dependence, reflecting an incompletely quenched orbital angular momentum. The near orbital degeneracy of the t_2g_ orbitals expected for these close-to-octahedral structures will also make the electronic *g*-factor very sensitive to vibronic couplings.^34^ This can contribute to the minor differences observed in the susceptibilities but, more importantly, will also provide a mechanism for very system dependent broadening of EPR signals. For temperature-coded data, see Supporting Information section S5 and Figures S15–S17.

The EPR spectra of [Fe(brphtmeimb)_2_]PF_6_, [Fe(meophtmeimb)_2_]PF_6_, and [Fe(coohphtmeimb)_2_]PF_6_ do not show any EPR signal at X-band frequencies, in either perpendicular or parallel mode (for details, see Supporting Information section S6), similar to their parent complex [Fe(phtmeimb)_2_]PF_6_.^23^

The low-temperature (80 K) Mößbauer spectra of [Fe(brphtmeimb)_2_]PF_6_, [Fe(meophtmeimb)_2_]PF_6_, and [Fe(coohphtmeimb)_2_]PF_6_ reveal a quadrupole split doublet with almost the same center shift (CS) and magnitude of the electric quadrupole splitting (QS) indicating low-spin Fe(III), just as the parent [Fe(phtmeimb)_2_]PF_6_ complex at 80 K (Supporting Information Figure S18).^23^ Furthermore, the 295 K spectrum of [Fe(meophtmeimb)_2_]PF_6_ also shows an extra doublet (spectral intensity 25(5)%) with CS and QS representative of low-spin Fe(II) (Supporting Information Figure S19). The Lorentzian line width Γ for the main Fe(III) component in the three spectra was only about 0.27(1) mm/s at 295 K, unveiling a narrow distribution in crystal environments in all samples. The spectra at low temperature show an asymmetric doublet structure with broad lines (Figure S18). The fitting results are presented in Table S6 which also includes the result from [Fe(phtmeimb)_2_]PF_6_.^23^ The Fe(II) doublet seen in the room temperature spectrum for [Fe(meophtmeimb)_2_]PF_6_ is hardly detected in the low-temperature (80 K) spectrum (Figure S18). An analysis shows a maximum spectral intensity of less than 3% for this Fe(II) component at around 80 K in the [Fe(meophtmeimb)_2_]PF_6_ sample. This could be due to different Mößbauer recoil free factors (*f*) for Fe(III) and Fe(II) in this complex. The *f*-factors become more equal at lower temperature, which is why a maximum limit of the ratio Fe(II)/Fe(III) to less than 3% can be determined. One explanation to the origin of the Fe(II) impurity(ies) is given in the caption of Supporting Information Figure S19. The center shift and electric quadrupole splitting for all of the doublets above fall in the range of reported values for low-spin *S* = ^1^/_2_, Fe(III) ions.^23,24^ In Supporting Information Figure S20, areas of different Fe valencies are presented in a |QS| vs CS (at 80 K) diagram for other Fe-carbenes. The asymmetries of the Fe(III) doublet found at 80 K can furthermore be explained on the basis of magnetic relaxation effects^35^ and a negative sign of QS. The relaxation time of the magnetic moment (in fact, the Mößbauer effect detects the magnetic hyperfine field acting at the Fe nucleus) of the Fe(III) ion at 80 K is comparable to the observation time τ of the Mößbauer effect. The observation time τ corresponds to the mean lifetime of the nuclear excited level, in the case of ^57^Fe spectroscopy to ∼70 ns. Magnetization, EPR, and Mößbauer results used to assign the spin state of the investigated compounds are summarized in Table 1.

**Table 1:** Magnetization, EPR, and Mößbauer Results Used to Assign the Spin State of the Investigated Compounds

Complex	Magnetization	EPR	Mößbauer (CS and QS), mm/s
[Fe(phtmeimb)_2_]^+^ ^23^	*S* = ^1^/_2_; *g* ≈ 2.00	No Signal	(−0.090 and 1.539);low spin *S* = ^1^/_2_
[Fe(brphtmeimb)_2_]^+^	*S* = ^1^/_2_; *g* ≈ 2.00	No Signal	(−0.081 and 1.666);low spin *S* = ^1^/_2_
[Fe(meophtmeimb)_2_]^+^	*S* = ^1^/_2_; *g* ≈ 2.00	No Signal	(−0.089 and 1.620);low spin *S* = ^1^/_2_
[Fe(coohphtmeimb)_2_]^+^	*S* = ^1^/_2_; *g* ≈ 2.00	No Signal	(−0.056 and 1.595);low spin *S* = ^1^/_2_

### Steady State Spectroscopy

**Figure 3:**
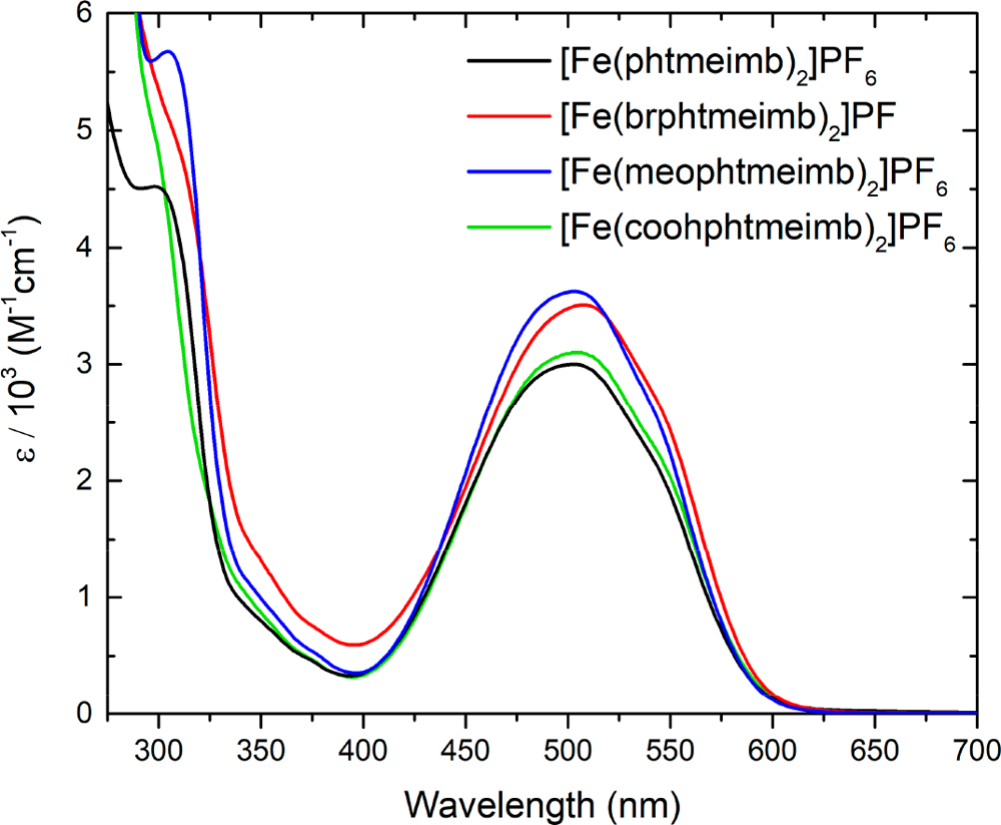
UV–vis absorption spectra of [Fe(phtmeimb)_2_]PF_6_,^23^ [Fe(brphtmeimb)_2_]PF_6_, [Fe(meophtmeimb)_2_]PF_6_, and [Fe(coohphtmeimb)_2_]PF_6_. All complexes were measured in acetonitrile except for [Fe(coohphtmeimb)_2_]PF_6_ which was measured in methanol (to enable higher concentration).

The steady state absorption spectra of [Fe(brphtmeimb)_2_]PF_6_, [Fe(meophtmeimb)_2_]PF_6_, and [Fe(coohphtmeimb)_2_]PF_6_ are very similar to the parent complex [Fe(phtmeimb)_2_]PF_6_ (Figure 3). No significant trend in the absorption maxima shift can be discerned with the given experimental accuracy. These only minor differences suggest that the para-substitution of the phenyl moiety has little impact on the excited state of [Fe(phtmeimb)_2_]PF_6_. In fact, one can suspect that phenyl rings are not involved in the transitions in the lower energy manifold. This notion is corroborated by the electrochemical data (see below). Therefore, the transition peaking at around 500 nm is assigned to an ^2^LMCT-band for all complexes, as already elucidated for the parent compound.^23^ The steady state absorption data for all complexes is collected in Table 2.

**Table 2:** Steady State Photophysical Properties Including the Absorption Maximum (Abs max), Its Full Width at Half-Maximum (FWHM), the Peak Extinction Coefficient (ε), the Emission Maximum (Em max), Its FWHM, the Emission Quantum Yield (ϕ), and Finally the 0–0 Energy (*E*_00_) of All Complexes Discussed in This Report

Substituent	Abs max^a^(nm/eV)	Abs fwhm (eV)	Ext coeffε (M^–1^ cm^–1^)	Em max^a^(nm/eV)	Em fwhm (eV)	ϕ (%)	*Ε*_00_ (eV)
[Fe(phtmeimb)_2_]PF_6_^b^	502/2.47	0.58	3000	655/1.89	0.43	2.1	2.14
[Fe(brphtmeimb)_2_]PF_6_	508/2.44	0.60	3500	658/1.88	0.43	1.8	2.13
[Fe(meophtmeimb)_2_]PF_6_	503/2.74	0.59	3600	658/1.88	0.43	1.7	2.13
[Fe(coohphtmeimb)_2_]PF_6_	505/2.46	0.58	3100	661/1.88	0.43	1.9	2.14

a Defined as where the derivative of the spectrum with respect to wavelength is zero.

b Data from ref (23).

**Figure 4:**
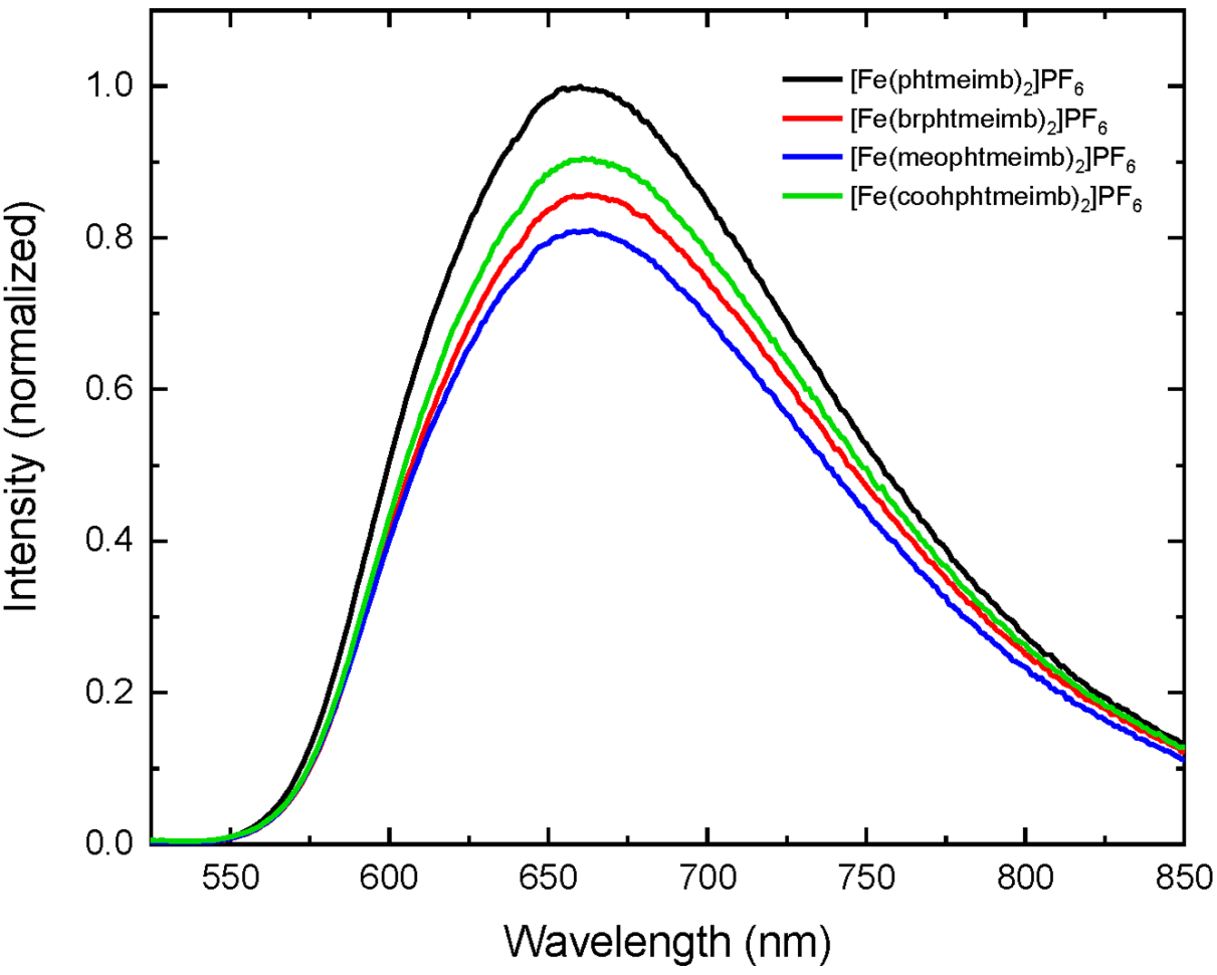
Emission spectra of [Fe(phtmeimb)_2_]PF_6_^23^ compared to [Fe(brphtmeimb)_2_]PF_6_, [Fe(meophtmeimb)_2_]PF_6_, and [Fe(coohphtmeimb)_2_]PF_6_. All complexes are dissolved in acetonitrile. The emission spectrum of [Fe(phtmeimb)_2_]PF_6_ has been normalized at its peak wavelength; others are scaled by their relative emission quantum yields.

The steady state emission spectra for [Fe(phtmeimb)_2_]PF_6_, [Fe(brphtmeimb)_2_]PF_6_, [Fe(meophtmeimb)_2_]PF_6_, and Fe(coohphtmeimb)_2_]PF_6_ are shown in Figure 4. After excitation at 502 nm, all complexes show a broad emission centered around 658 nm, nearly identical to what was observed for the parent complex, [Fe(phtmeimb)_2_]PF_6_.^23^ To further investigate the nature of the emission, excitation spectra were recorded for [Fe(brphtmeimb)_2_]PF_6_, [Fe(meophtmeimb)_2_]PF_6_, and [Fe(coohphtmeimb)_2_]PF_6_. The emitted intensity probed at 625 nm as a function of excitation wavelength agrees for all three complexes reasonably with their absorption spectra (see Figures S21–S23), which is an indication that the emission indeed originates from the excited ^2^LMCT state of the complex. The para-substituted complexes tend to have a slightly lower emission quantum yield compared to the parent complex. Based on comparative measurements to the 2.1% quantum yield reference that was reported for [Fe(phtmeimb)_2_]PF_6_,^23^ the quantum yield of [Fe(brphtmeimb)_2_]PF_6_ is 1.8%, that of [Fe(meophtmeimb)_2_]PF_6_ is 1.7%, and that of [Fe(coohphtmeimb)_2_]PF_6_ is 1.9%. The data for all complexes is collected in Table 2.

### Cyclic Voltammetry and Spectroelectrochemistry

**Figure 5:**
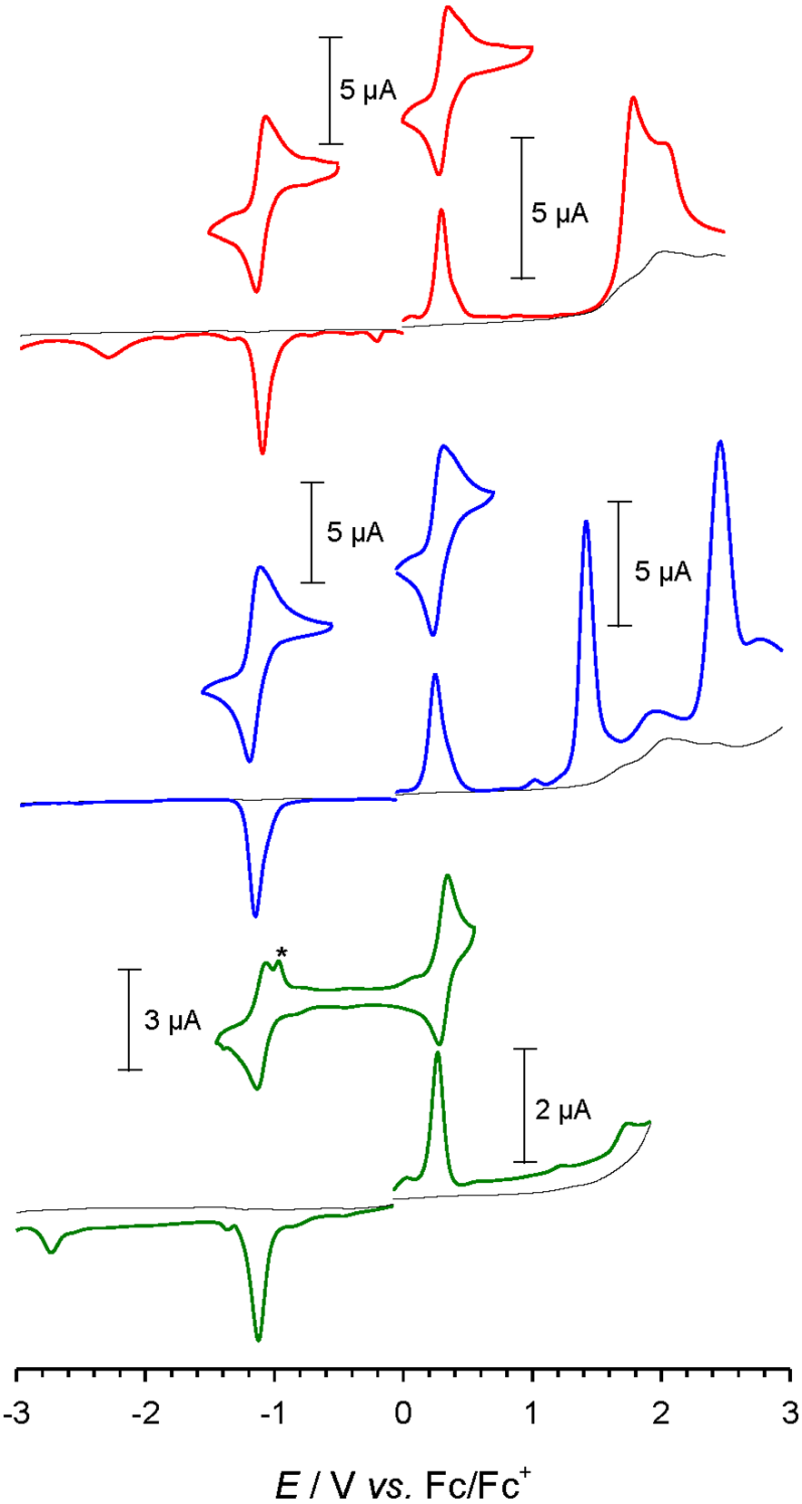
Differential pulse and cyclic voltammograms of [Fe(brphtmeimb)_2_]PF_6_ (1 mM, 0.1 V s^–1^, red line) (top), [Fe(meophtmeimb)_2_]PF_6_ (1.2 mM, 0.1 V s^–1^, blue line) (middle), and [Fe(coohphtmeimb)_2_]PF_6_ (0.6 mM, 0.05 V s^–1^, green line, * postpeak due to adsorption of [Fe(coohphtmeimb)_2_] on the electrode) (bottom) in acetonitrile (0.1 M N(*n*-butyl)_4_PF_6_, black line).

The voltammetric characterization of [Fe(brphtmeimb)_2_]PF_6_, [Fe(meophtmeimb)_2_]PF_6_, and [Fe(coohphtmeimb)_2_]PF_6_ (Figure 5 and Table 3) revealed two reversible one-electron waves that can attributed to the Fe(III/II) and Fe(IV/III) couples in analogy to the parent complex [Fe(phtmeimb)_2_]PF_6_.^23^ In comparison to the latter, the potentials of the metal-centered couples show only very moderate shifts of about 30 mV toward higher potentials for the electron-withdrawing bromide and carboxylic acid substituents and similar shifts in the opposite direction for the electron-donating methoxy substituent. Analogous but more pronounced substituent effects were found for the potential of the first ligand oxidation that is shifted relative to the parent complex by +80 and −280 mV in [Fe(brphtmeimb)_2_]PF_6_ and [Fe(meophtmeimb)_2_]PF_6_, respectively. Further reduction of the Fe(II) state was observed for [Fe(brphtmeimb)_2_]PF_6_ and [Fe(coohphtmeimb)_2_]PF_6_. The peaks at −2.3 and −2.7 V, respectively, can be tentatively attributed to the reduction of the functionalized aryl moieties rather than the actual carbene ligands. The latter are not reduced within the available potential window in the case of the parent complex and [Fe(meophtmeimb)_2_]PF_6_, and spectroscopic data confirms that the same situation also applies to [Fe(brphtmeimb)_2_]PF_6_ and [Fe(coohphtmeimb)_2_]PF_6_.

**Figure 6:**
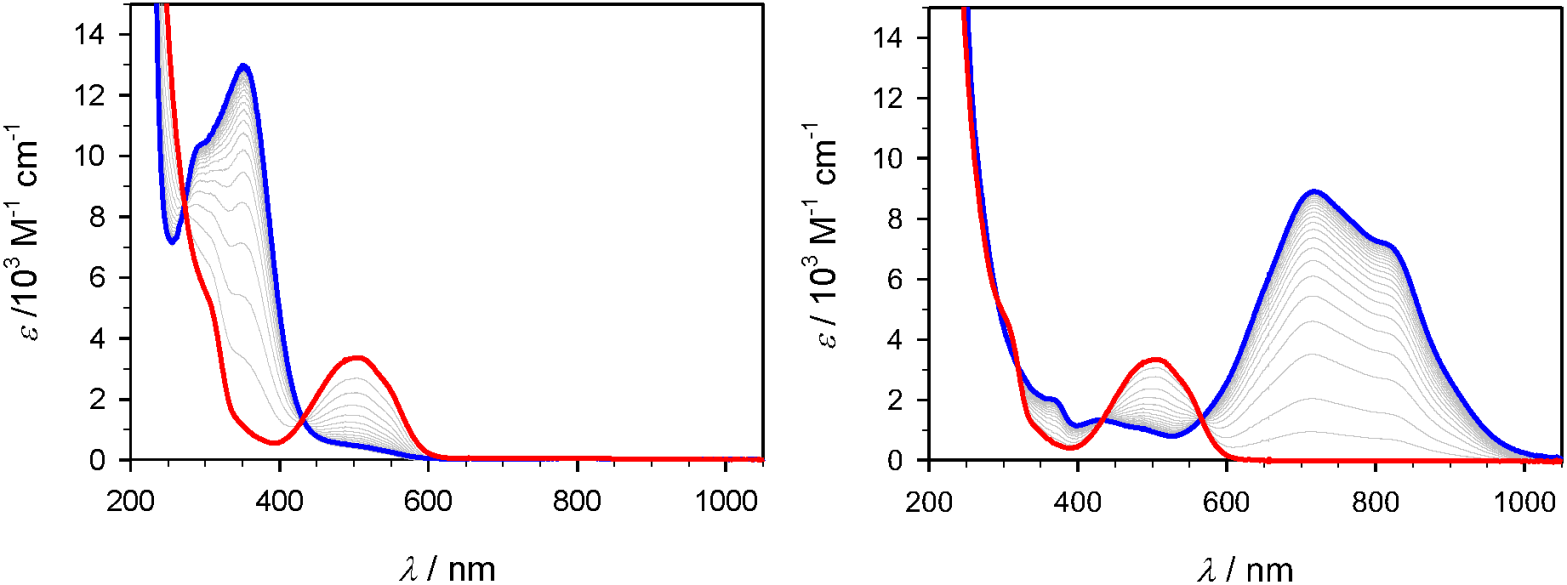
UV–vis spectroelectrochemistry of [Fe(brphtmeimb)_2_]PF_6_ (red line) in acetonitrile (0.1-M N(*n*-butyl)_4_PF_6_). Left: Reduction at −1.44 V generating [Fe(brphtmeimb)_2_] (blue line). Right: Oxidation at 0.76 V generating [Fe(brphtmeimb)_2_]^2+^ (blue line).

**Figure 7:**
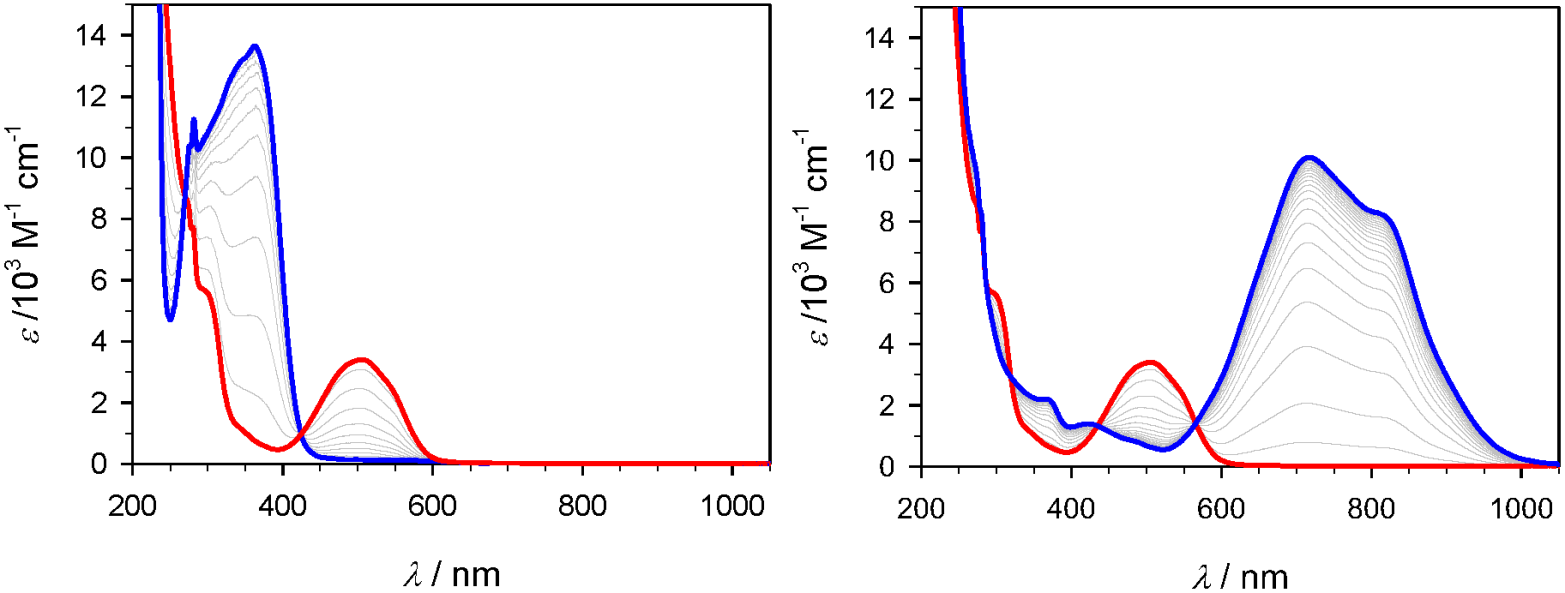
UV–vis spectroelectrochemistry of [Fe(meophtmeimb)_2_]PF_6_ (red line) in acetonitrile (0.1 M N(*n*-butyl)_4_PF_6_). Left: Reduction at −1.44 V generating [Fe(meophtmeimb)_2_] (blue line). Right: Oxidation at 0.76 V generating [Fe(meophtmeimb)_2_]^2+^ (blue line).

**Figure 8:**
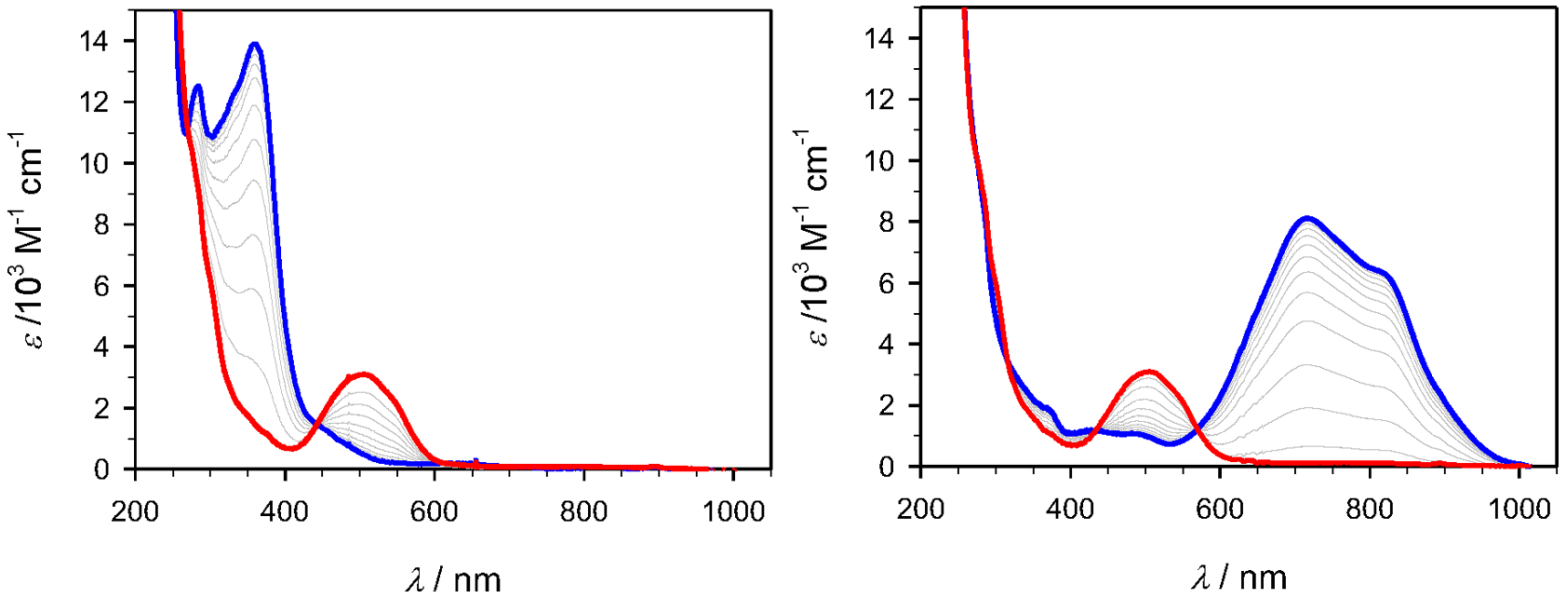
UV–vis spectroelectrochemistry of [Fe(coohphtmeimb)_2_]PF_6_ (red line) in acetonitrile (0.1 M N(*n*-butyl)_4_PF_6_). Left: Reduction at −1.44 V generating [Fe(coohphtmeimb)_2_] (blue line). Right: Oxidation at 0.76 V generating [Fe(coohphtmeimb)_2_]^2+^ (blue line).

Spectroelectrochemistry data featuring the Fe(III) ground state together with the corresponding Fe(II) and Fe(IV) states obtained from controlled potential bulk electrolysis is shown in Figures 6–8 for [Fe(brphtmeimb)_2_]PF_6_, [Fe(meophtmeimb)_2_]PF_6_, and [Fe(coohphtmeimb)_2_]PF_6_. The data is summarized in Table 3. For the Fe(III) and Fe(IV) complexes, the energies of their lowest energy LMCT bands are within error margins indistinguishable from those of the parent complex (Table 3).^23^ At a first glance, this appears to be at odds with the trends in electrochemical potentials, in particular the significantly lowered potential for ligand oxidation of [Fe(meophtmeimb)_2_]PF_6_ in combination with the marginal effects on the metal couples. Also, the MLCT bands of the Fe(II) complexes are rather similar in energy. This places the potentials for ligand reduction involved in the electronic excitation in all cases well below the lower limit of the potential window, while electrochemical reduction of [Fe(brphtmeimb)_2_]^*n*+^ can be observed already at −2.3 V. These results support the notion that the observed electrochemical ligand oxidation and reduction processes are not involved in the spectroscopic transitions if they are essentially localized on the phenyl rings as one might anticipate in particular for ligand oxidation of [Fe(meophtmeimb)_2_]^*n*+^ and ligand reduction of [Fe(brphtmeimb)_2_]^*n*+^.

**Table 3:** Electrochemical and UV–vis Spectroscopic Data^a^

	*E°*/V			λ_max_/nm(ε/10^3^ M^–1^ cm^–1^)		
Complex	Fe(III/II)^b^	Fe(IV/III)^b^	*L*_ox_^c^	*n* = 0(Fe(II), MLCT)	*n* = 1(Fe(III), LMCT)	*n* = 2(Fe(IV), LMCT)
[Fe(phtmeimb)_2_]^*n*+^	–1.16	0.26	1.67	348 (10.8)	502 (3.0)	715 (6.8)
[Fe(brphtmeimb)_2_]^*n*+^	–1.14	0.28	1.75	352 (12.8)	504 (3.3)	715 (8.9)
[Fe(meophtmeimb)_2_]^*n*+^	–1.19	0.24	1.39	363 (13.5)	505 (3.3)	716 (10.1)
[Fe(coohphtmeimb)_2_]^*n*+^	–1.13	0.29	>1.9	361 (13.9)	505 (3.1)	717 (8.1)

a In acetonitrile with 0.1 M N(*n*-butyl)_4_PF_6_ vs Fc.

b Half-wave potential (CV).

c Peak potential (DPV).

### Transient Absorption (TA) Spectroscopy

**Figure 9:**
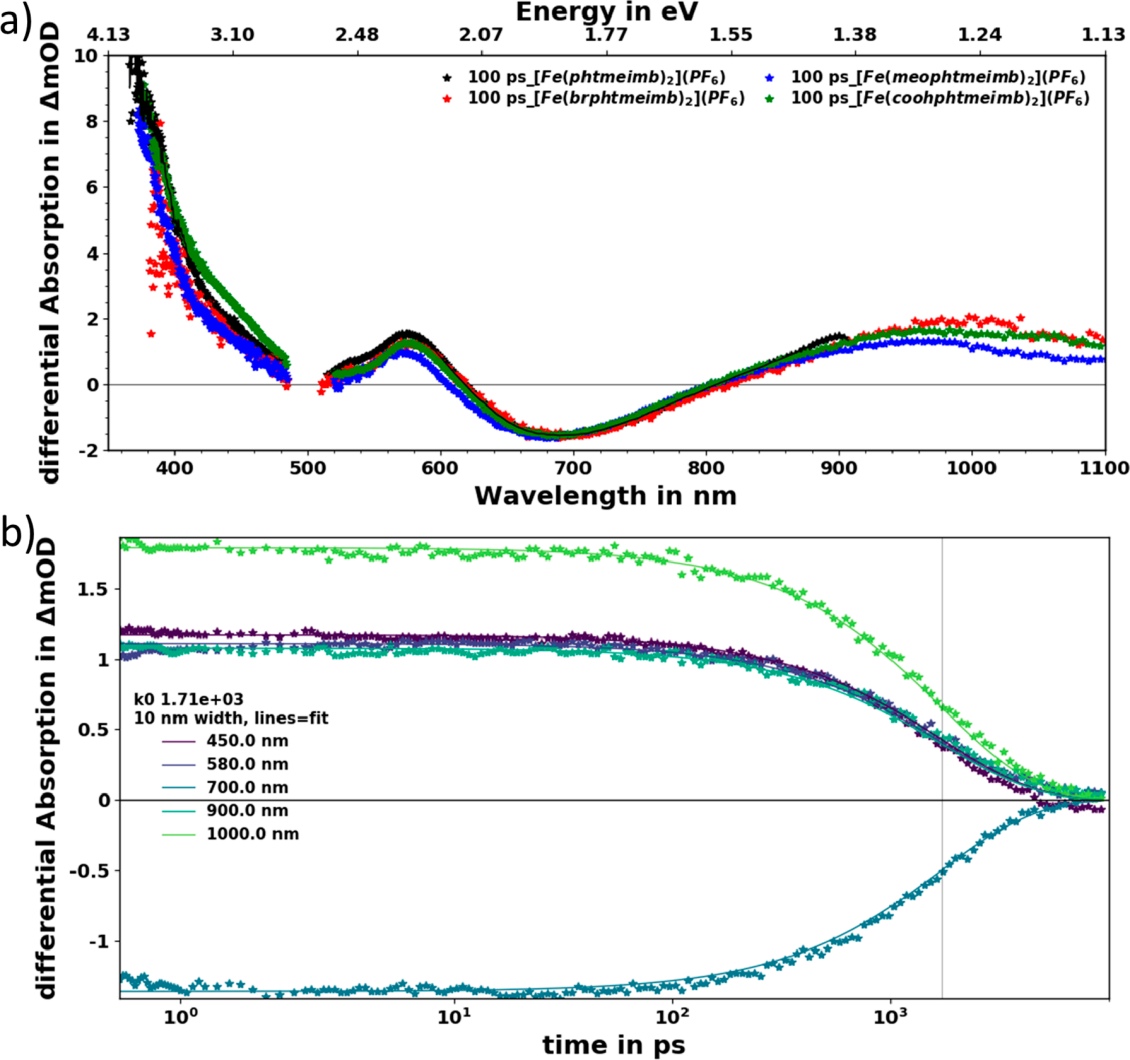
(a) Measured TA spectra at 100 ps of [Fe(phtmeimb)_2_]PF_6_, [Fe(brphtmeimb)_2_]PF_6_, [Fe(meophtmeimb)_2_]PF_6_, and [Fe(coohphtmeimb)_2_]PF_6_ in acetonitrile compared. The data has been chirp and background corrected and cut to remove excitation (∼500 nm) scatter. (b) Kinetics at selected wavelengths of [Fe(brphtmeimb)_2_]PF_6_, also including the single exponential fit from global analysis (measured data is shown as symbols and the fit as solid lines). The kinetics for the other complexes are shown in Supporting Information Figures S25 and S26.

The transient absorption (TA) spectra of [Fe(phtmeimb)_2_]PF_6_, [Fe(brphtmeimb)_2_]PF_6_, [Fe(meophtmeimb)_2_]PF_6_, and [Fe(coohphtmeimb)_2_]PF_6_ in acetonitrile recorded 100 ps after excitation in the ^2^LMCT band at ∼500 nm are shown in Figure 9a. The TA spectra of all complexes share the same spectral features, and the selected time delay is representative for showing the fully developed spectra that later only decay. At 500 nm, the ground state bleach (GSB) region is overwhelmed by excited state absorption (ESA). The pronounced ESA below 450 nm is in line with the LMCT assignment and the corresponding absorption of the Fe(II) ground state (see Figures 6–8). Additional ESA with a peak around 580 nm and the broad absorption in the red and near-infrared region can be attributed to the NHC ligand radical with the superimposed stimulated emission signal peaking around 700 nm. The stimulated emission dynamics unambiguously reports on the evolution of the emissive excited state population. For [Fe(brphtmeimb)_2_]PF_6_, the selected kinetics at the before mentioned wavelengths are shown in Figure 9b; all kinetics follow the same exponential decay. The kinetics for [Fe(meophtmeimb)_2_]PF_6_ and [Fe(coohphtmeimb)_2_]PF_6_ are similar (see Supporting Information Figures S24 and S25). The excited state decay can be accurately described by a single exponential model and a global fit^36^ to the data resulting in a universal lifetime of ∼2 ns at all observed features and for all [Fe(phtmeimb)_2_]PF_6_ derivatives; see Table 4. These results are in good agreement with emission lifetimes (of 1.9 ns) determined by time-correlated single photon counting (TC-SPC, in Supporting Information section S10, Figure S27, and Table S7). The excited state lifetimes of all three derivatives are thus very similar to the 2 ns previously reported for the parent complex [Fe(phtmeimb)_2_]PF_6_.^23^

**Table 4:** Collected Photophysical Properties Including the Emission Quantum Yield (φ), Excited State Lifetime (τ), Radiative Decay Rate (*k*_r_), and Non-Radiative Decay Rate (*k*_nr_) of All Complexes Discussed in This Report

Complex	ϕ (%)	τ (ns)	*k*_r_(10^7^ s^–1^)	*k*_nr_(10^8^ s^–1^)
[Fe(phtmeimb)_2_]PF_6_ ^23^	2.1	2.0	1.1	5.0
[Fe(brphtmeimb)_2_]PF_6_	1.8	1.7	1.1	5.8
[Fe(meophtmeimb)_2_]PF_6_	1.7	1.7	1.0	5.8
[Fe(coohphtmeimb)_2_]PF_6_	1.9	1.6	1.2	6.1

Based on the quantum yield and the lifetime of the excited state, the substituents of [Fe(phtmeimb)_2_]PF_6_ have very minor effects on both radiative and nonradiative decay pathways (Table 4). (The nonradiative decay rate is increased from 5.0 × 10^8^ s^–1^ ([Fe(phtmeimb)_2_]PF_6_) to ∼6 × 10^8^ s^–1^ for the substituted complexes.) Since the energetic positions of the MC states are very similar for all complexes (based on quantum chemical calculations, see Supporting Information section S11), the accelerated nonradiative decay of the substituted complexes could be due to faster internal conversion directly from the ^2^LMCT excited state to the ground state. The change in photophysical properties, however, is only minor, which means that introducing substituents to the [Fe(phtmeimb)_2_]PF_6_ framework still preserves strong photoluminescence from the ^2^LMCT state with an ∼2 ns lifetime.

### Quantum Chemical Calculations

**Figure 10:**
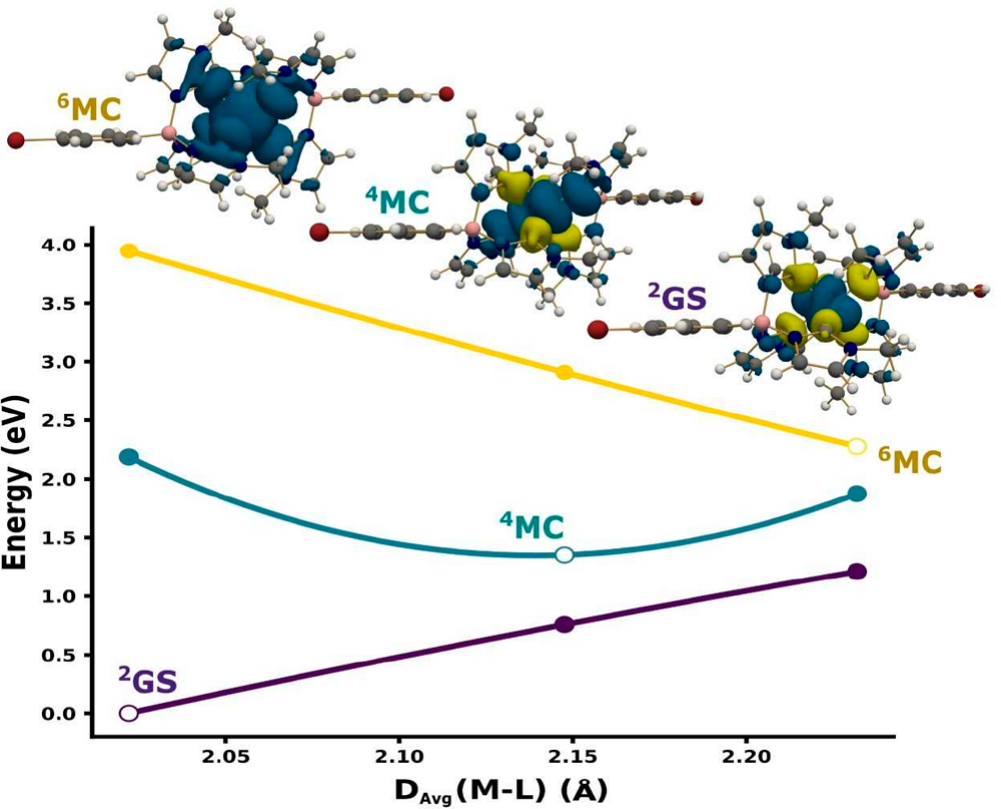
Energy graphical representation of the [Fe(brphtmeimb)_2_]PF_6_ doublet ground state (^2^GS), metal center quartet state (^4^MC), and metal center hextet state (^6^MC) along the internal coordinate metal–ligand averaged bond distance (*D*_avg_(M–L)). The energies of the relaxed geometries in the doublet, quartet, and hextet multiplicities are represented by empty points, while filled points correspond to the point energies calculated at the corresponding structures. The calculated spin densities for ^2^[Fe(brphtmeimb)_2_]^+^, ^4^[Fe(brphtmeimb)_2_]^+^, and ^6^[Fe(brphtmeimb)_2_]^+^ are also displayed. All calculated values are given in Supporting Information Tables S8–S12.

Key points on the potential energy surfaces of [Fe(phtmeimb)_2_]^+^ as well as [Fe(brphtmeimb)_2_]^+^, [Fe(meophtmeimb)_2_]^+^, and [Fe(coohphtmeimb)_2_]^+^ congeners were calculated by using unrestricted density functional theory (DFT). For brevity and due to the close similarity between the different studied molecules, only the [Fe(brphtmeimb)_2_]^+^ energy profile has been plotted in Figure 10. The quantum chemical results reveal a doublet ground state (^2^GS) and quartet (^4^MC) and hextet states (^6^MC) stable under phenyl group functionalization following the same energy trend as previously reported for [Fe(phtmeimb)_2_]^+^.^23^ The calculated spin density for the doublet ground state of all the investigated complexes is found to be mainly located on the metal and carbene lone pairs, as shown for [Fe(brphtmeimb)_2_]^+^ in Figure 10. Overall, the results from the quantum chemical calculations highlight the similarity of the electronic structure properties across the full series of complexes, including the lack of involvement of the phenyl-based moieties, which is consistent with the overall observed lack of electronic communication between the metal center and the side groups. The spin density on the metal in the relaxed quartet and hextet states indicates the same metal center nature of these states for the three iron carbene derivatives. All spin densities for [Fe(phtmeimb)_2_]^+^ and congeners are displayed in Supporting Information Table S8. The relaxed ground state geometries of the three iron complexes are in good agreement with the reported X-ray structures. The average iron–carbene distances are also reported in Supporting Information Tables S8–S12 for all complexes and suggest unremarkable structural changes due to addition to bromide, methoxy, or carboxylic groups in the 4-position of the phenyl.

## Conclusion

In conclusion, for the triad [Fe(brphtmeimb)_2_]PF_6_, [Fe(meophtmeimb)_2_]PF_6_, and [Fe(coohphtmeimb)_2_]PF_6_ that are building on the parent compound [Fe(phtmeimb)_2_]PF_6_ containing the scorpionate ligand [phtmeimb]^−^, the substitution of the latter in the 4-phenyl position with either −Br, −OMe, or −COOH substituents did not result in any significant changes of the ground state properties such as geometry and magnetic properties, however adding three new iron complexes with ns lifetime and visible photoluminescence to the existing very small library of such complexes. Electrochemistry and quantum chemistry calculations indicate weak electronic communication between the phenyl moiety of the scorpionate ligand and the iron center, leading to only marginal electrochemical shifts between the complexes. The essentially identical charge-transfer absorption bands of the three complexes in their Fe(II), Fe(III), and Fe(IV) states, further suggest that the spectroscopically relevant ligand orbitals do not extend over the phenyl moieties. Importantly, the ^2^LMCT excited state of the substituted Fe(III) complexes not only retains the excited state energy but also shows only modestly reduced emission quantum yields and excited state lifetimes relative to the parent complex. This demonstrates that the favorable photophysical properties, characteristic of the parent complex, could be exploited in prospective photoactive assemblies with the 4-phenyl position as an attachment point. Our results reveal remarkably small effects of both electron-withdrawing and -donating substituents on the ground and excited state properties, thereby demonstrating that the [Fe(phtmeimb)_2_]PF_6_ motif should tolerate a wide range of modification for the above purposes without loss of the favorable photofunctionalities.

## Supporting Information Available

Synthesis, ^1^H and ^13^C NMR spectra, HR-MS spectra, single crystal X-ray diffraction, magnetic susceptibility and magnetization measurements, Mößbauer spectroscopy, electron paramagnetic resonance measurements, steady state spectroscopy, steady state absorption, steady state emission, transient absorption spectroscopy, TCSPC data, and quantum chemistry, including figures and tables (PDF)
