# Supplementary material for: Photophysical Integrity of the Iron(III) Scorpionate Framework in Iron(III)–NHC Complexes with Long-Lived 2LMCT Excited States
Source: Inorg Chem. 2022 Oct 24;61(44):17515–26. doi: 10.1021/acs.inorgchem.2c02410 (PMC9644379; doi:10.1021/acs.inorgchem.2c02410)
Supplement: Supplementary file 1 — ic2c02410_si_001.pdf [file ic2c02410_si_001.pdf]

SUPPORTING INFORMATION

Photophysical Integrity of the Iron(III) Scorpionate Framework in Iron(III)-NHC Complexes with Long-lived ^{2}LMCT Excited States

Om Prakash,[a] Linnea Lindh,[b, c] Nidhi Kaul,[d] Nils W. Rosemann,[b] Iria Bolaño Losada,[c] Catherine Johnson,[d] Pavel Chábera,[b] Aleksandra Ilic,[a] Jesper Schwarz,[a] Arvind Kumar Gupta,[a] Jens Uhlig,[b] Tore Ericsson,[e] Lennart Häggström,[e] Ping Huang,[d] Jesper Bendix,[f] Daniel Strand,[a] Arkady Yartsev,[b,] Reiner Lomoth,[d,]* Petter Persson,[c,]* Kenneth Wärnmark[a,]**

Corresponding Authors

*A.Y.: E-mail: arkady.yartsev@chemphys.lu.se.

*R.L.: E-mail: reiner.lomoth@kemi.uu.se.

*P.P.: E-mail: petter.persson@teokem.lu.se.

*K.W.: E-mail: kenneth.warnmark@chem.lu.se.

List of contents **Page**

S1. Synthesis……………………………………………………………………………. S3

S1.1 General experimental details

S1.2 Synthesis of [Fe(brphtmeimb)$_2$]PF$_6$

S1.3 Synthesis of [Fe(meophtmeimb)$_2$]PF$_6$

S1.4 Synthesis of [Fe(coohphtmeimb)$_2$]PF$_6$

S2. ^{1}H and ^{13}C{^{1}H} NMR Spectra……………………………………………………… S9

S3. HR-MS Spectra……………………………………………………………………….. S19

S4. Single Crystal X-ray diffraction……………………………………………………… S22

S5. Magnetic susceptibility and magnetization measurements………………………… S29

S6. Electron paramagnetic resonance measurements………………………………… S32

S7. Mößbauer spectroscopy……………………………………………………………. S34

S8. Steady State Spectroscopy…………………………………………………………. S37

S8.1 Steady state absorption spectroscopy

S8.2 Steady state emission spectroscopy

S9. Transient absorption spectroscopy……………………………………………….... S42

S10. Time-correlated single photon counting……………………………………………. S45

S11. Quantum Chemistry………………………………………………………………. S47

S12. References…………………………………………………………………………... S51

S1. Synthesis

S1.1 General experimental details

^{1}H and ^{13}C NMR spectra were recorded on a Bruker Avance II 400 MHz NMR spectrometer. Chemical shifts (δ) are reported to the shift-scale calibrated with the residual NMR solvent; CD_3CN (1.94 ppm for ^{1}H NMR spectra). Electrospray ionization–high resolution mass spectrometry (ESI–HRMS) and atmospheric pressure chemical ionization (APCI) for mass spectrometry were recorded on a Waters Micromass Q-Tof micro mass spectrometer. Infrared spectra were recorded as the neat compound on a Bruker Alpha-P FTIR spectrometer. Melting points of the compounds were measured on a Stuart Scientific Melting Point Apparatus-SMP3. Elemental analyses were performed by Mikroanalytisches Laboratorium KOLBE (Mülheim an der Ruhr, Germany). Size of the bio-beads column is 120 cm in length and 4.5 cm in width.

Potassium *tert*-butoxide (1 M solution in THF), *n*-BuLi (2.5 M solution in THF), 1-bromo-4-trimethylsilyl-benzene, (4-methoxyphenyl)trimethylsilane, boron tribromide (1M solution in DCM), 1-methylimidazole, K_2CO_3, and methyl iodide were purchased from Aldrich. Anhydrous ferrous chloride ($FeCl_2$) and potassium hexafluorophosphate (KPF_6) were purchased from Acros Organics. THF (Honeywell) was dried over Na/benzophenone and was subsequently distilled under argon prior to use. Anhydrous CH_3CN, DMF, methanol, and dichloromethane, diethyl ether was obtained from a PureSolv PSM-768 and Braun SPS-800 system respectively.

S1.2 Synthesis of $[Fe(brphtmeimb)_2]PF_6$

[(4-Bromophenyl)tris(3-methyl-1H-imidazol-3-ium-1-yl)borate] bis(hexaflurophosphate)

$[brphtmeimbH_3](PF_6)_2$

A mixture of 1-bromo-4-trimethylsilyl-benzene (2.291 g, 10.0 mmol) and boron tribromide (1 M solution in DCM, 10.1 mL, 10.1 mmol) was heated to 70 °C in an ace pressure tube for 20 h. After cooling reaction mixture to room temperature, 1-methylimidazole (2.43 mL, 30.5 mmol) was quickly added under N_2 flow and the reaction mixture was further heated to 80 °C for additional 20 h. The reaction mixture was cooled to room temperature and the resulting precipitate was collected by filtration using a sintered glass funnel (porosity #4). The precipitate was washed with dry acetone to give product as a white powder. The white powder was dissolved in distilled water, reprecipitated by addition of aqueous potassium hexafluorophosphate (25.0 mmol) and collected by filtration. The white precipitate washed thoroughly with water. After extraction with methanol, the resulting pure compound was dried under vacuum to yield a white powder (4.210 g, Yield: 60%). ^{1}H NMR (400 MHz, CD_3CN, 5 mM): δ (ppm) 7.99 (s, 3H, H_{Im-g}), 7.63-7.60 (m, 2H, H_{Ph-c}), 7.44 (m, 3H, H_{Im-d}), 7.10 (m, 3H, H_{Im-e}), 7.08-7.06 (m, 2H, H_{Ph-b}), 3.80 (s, 9H, H H_{Im-f}); ^{13}C{^{1}H} NMR (100.3 MHz, CD_3CN, 5 mM): δ (ppm) 140.4 (C_{Im-6}), 135.8 (C_{Ph-c}), 132.5 (C_{Ph-b}), 125.7 (C_{Ph-a}), 124.8 (C_{Im-e}), 124.5 (C_{Im-d}), 36.48 (C_{Im-f}). ESI–HRMS (m/z): $[(C_{18}H_{22}N_6BrB)+(PF_6)]^+$ calcd for $C_{18}H_{22}N_6BBrPF_6$, 559.0804; found, 559.0807; Elemental analysis: (% calcd, % found for $C_{18}H_{22}N_6P_2BBrF_{12}$): C(30.75, 30.78), H(3.15, 3.16), N(11.95, 11.92).

[(4-Bromopheny)ltris(3-methylimidazol-2-ylidene)borate]iron hexafluorophosphate

[Fe(brphtmeimb)$_2$]PF$_6$

A mixture of [(4-bromophenyl)tris(3-methyl-1*H*-imidazol-3-ium-1-yl)borate] bis(hexafluorophosphate) (0.351 g, 0.5 mmol) and anhydrous $FeCl_2$ (0.032 g, 0.25 mmol) was dried under vacuum at 80 °C overnight. Dry DMF (7 mL) was charged under N_2. *t*-BuOK (2.0 mL, 2.0 mmol, 1 M in THF) was added dropwise at room temperature. The resulting reaction mixture was stirred for 30 min at rt. Aqueous KPF_6 solution was added to precipitate red solid which was filtered using a sintered glass funnel (porosity #4) and washed with water. The resulting red residue was extracted with CH_2Cl_2 (2 × 30 mL) and evaporated to dryness *in vacuo*. The resulting rose-red residue was dissolved in 5 mL of acetonitrile and the product was precipitated by addition of dry diethyl ether (50 mL). The rose-red precipitate was dissolved in a minimum amount of CH_3CN. The solution was filtered through a syringe filter w/ 0.2 μm PTFE membrane, and the compound was purified on a Bio-Beads S-X1 size-exclusion chromatography column. The product was eluted using CH_3CN/toluene (1:1) as the eluent. This was repeated twice. The fraction containing the product was evaporated to dryness under vacuum and the resulting residue was recrystallized from dry CH_2Cl_2 via slow diffusion of dry diethyl ether in the dark to yield rose-red crystals (0.076 g, 30%). ^{1}H NMR (400 MHz, CD_3CN, 25 mM): δ(ppm) 14.80 (m, 4H, $H_{Ph\text{-}c}$), 10.62 ((m, 4H, $H_{Ph\text{-}b}$), 4.96 (s, 18H, $H_{Im\text{-}f}$), 1.36 (s, 6H, $H_{Im\text{-}d}$), -12.50 (s, 6H, $H_{Im\text{-}e}$); ^{13}C{^{1}H} NMR (100.3 MHz, CD_3CN, 25 mM): δ (ppm) 141.6 ($C_{Ph\text{-}c}$), 134.7 ($C_{Ph\text{-}b}$), 125.0 ($C_{Ph\text{-}a}$), 47.3 ($C_{Im\text{-}d}$), 12.9 ($C_{Im\text{-}e}$), -30.3 ($C_{Im\text{-}f}$); IR (thin layer film) ν (cm^{-1}) 1562, 1445, 1413, 1340, 1290, 1272, 1179, 1122, 1079, 1031, 882, 831, 794, 733, 710, 558; MP: 350°C; ESI–HRMS (m/z): $[(C_{36}H_{38}Br_2N_{12}B_2Fe)-(PF_6)]^+$ calcd for $C_{36}H_{38}Br_2N_{12}B_2Fe$, 874.1245; found, 874.1249; Elemental analysis: (% calculated, % found for $C_{36}H_{38}N_{12}PBr_2B_2F_6Fe$): C (42.35, 42.48), H (3.75, 3.73), N (16.46, 16.25).

S1.3 Synthesis of [Fe(meophtmeimb)$_2$]PF$_6$

[(4-Methoxyphenyl)tris(3-methyl-1H-imidazol-3-ium-1-yl)borate] bis(hexafluorophosphate)

[meophtmeimbH$_3$](PF$_6$)$_2$

A mixture of (4-methoxyphenyl)trimethylsilane (10.0 mmol) and boron tribromide (1 M solution in DCM, 10.0 mL, 10.0 mmol) was heated to 70 ^{o}C in an ace pressure tube for 20 h. After cooling reaction mixture to room temperature, 1-methylimidazole (2.43 mL, 30.5 mmol) was quickly added to the reaction mixture under N_2 flow and the reaction mixture was heated to 80 °C for another 20 h. After completion, the reaction mixture cooled to room temperature and resulting precipitate was collected by filtration using a sintered glass funnel (porosity #4). The collected precipitate was washed with dry acetone to give the pale-white powder. The powder was dissolved in distilled water, precipitated by addition of aqueous potassium hexafluorophosphate (25.0 mmol) and the resulting precipitate was collected by filtration before washing with water. The resulting compound was extract in methanol and dried under vacuum to yield the product as a pale-white powder (5.04 g, Yield: 77%). ^{1}H NMR (400 MHz, CD$_3$CN, 5 mM): δ (ppm) 8.00 (s, 3H, H_{Im-g}), 7.44-7.43 (m, 3H, H_{Im-d}), 7.12-7.11 (m, 3H, H_{Im-e}), 7.10-7.08 (m, 2H, H_{Ph-c}), 6.99-6.97(m, 2H, H_{Ph-b}), 3.82 (s, 9H, H_{Im-f}); ^{13}C{^{1}H} NMR (100.3 MHz, CD$_3$CN, 5 mM): δ (ppm) 139.2 (C_{Im-g}), 134.9 (C_{Ph-c}), 131.6 (C_{Ph-b}), 124.8 (C_{Ph-a}), 123.8 (C_{Im-e}), 123.6 (C_{Im-d}), 35.5 (C_{Im-f}); ESI–HRMS (m/z): $[(C_{19}H_{25}N_6OBPF_6)+(PF_6)]^+$ calcd for $C_{19}H_{25}N_6OBPF_6$, 509.1825; found, 509.1829; Elemental analysis: (% calcd, % found for $C_{19}H_{25}N_6OBP_2F_{12}$): C(34.88, 34.82), H(3.85, 3.90), N(12.85, 12.92).

[(4-methoxyphenyl)tris(3-methylimidazol-2-ylidene)borate]iron hexafluorophosphate

[Fe(meophtmeimb)$_2$]PF$_6$

A mixture of [(4-methoxyphenyl)tris(3-methyl-1*H*-imidazol-3-ium-1-yl)borate] bis(hexafluorophosphate) (0.654 g, 1.0 mmol) and anhydrous $FeCl_2$ (0.063 g, 0.55 mmol) was dried under vacuum at 80 °C overnight in a Schlenk tube connected to the house vacuum line. Dry DMF (7 mL) was charged under N_2. *t*-BuOK (3.5 mL, 3.5 mmol, 1 M in THF) was added dropwise and the resulting reaction mixture was stirred for 30 min at rt. Aqueous KPF_6 solution was added to precipitate the red solid which was filtered using a sintered glass funnel (porosity #4) and washed with water. The resulting red residue was extracted with CH_2Cl_2 (2 × 50 mL) and evaporated to dryness *in vacuo*. The resulting rose-red residue was dissolved in 5 mL of acetonitrile and the product was precipitated by the addition of dry diethyl ether (50 mL). The precipitate was recrystallized from dry CH_3CN via slow diffusion of dry diethyl ether in the dark to yield rose-red crystals (0.130 g, yield 28 %).; ^{1}H NMR (400 MHz, CD_3CN, 25 mM): δ(ppm) 14.55 (m, 4H, $H_{Ph\text{-}c}$), 9.92 (m, 4H, $H_{Ph\text{-}b}$), 5.25 (s, 6H, $H_{Ph\text{-}h}$), 4.91 (s, 18H, $H_{Im\text{-}f}$), 1.68 (s, 6H, $H_{Im\text{-}d}$), -12.25 (s, 6H, $H_{Im\text{-}e}$) ; ^{13}C{^{1}H} NMR (100.3 MHz, CD_3CN, 25 mM): δ (ppm) 163.3 ($C_{Ph\text{-}a}$), 141.6 ($C_{Ph\text{-}b}$), 57.2 ($C_{Ph\text{-}h}$), 49.7 ($C_{Im\text{-}d}$), 13.1 ($C_{Im\text{-}e}$), -28.4 ($C_{Im\text{-}f}$); IR (thin layer film) ν (cm^{-1}) 1558, 1444, 1407, 1348, 1299, 1270, 1174, 1110, 1079, 1029, 887, 830, 790, 739, 702, 553; MP 350 ˚C;ESI–HRMS (m/z): $[(C_{38}H_{44}O_2N_{12}B_2Fe)-(PF_6)]^+$ calcd for $C_{38}H_{44}O_2N_{12}B_2Fe$, 778.3246; found, 778.3265; Elemental analysis: (% calculated, % found for $C_{38}H_{44}N_{12}PB_2F_6O_2Fe\cdot0.25CH_2Cl_2$): C (48.64, 48.70), H (4.75, 4.74), N (17.80, 17.92).

S1.4 Synthesis of [Fe(coohphtmeimb)$_2$]PF_6

[(4-carboxyphenyl)tris(3-methylimidazol-2-ylidene)borate]iron hexafluorophosphate

[Fe(coohphtmeimb)$_2$]PF_6

[Fe(brphtmeimb)$_2$]PF_6 (0.120 g, 0.12 mmol) was dried at 80 °C in a Schlenk flask under vacuum overnight. Anhydrous THF (20 mL) was charged to the Schlenk flask at room temperature affording a red suspension. The reaction mixture was then cooled to -78 °C under N_2 atmosphere. *n*-BuLi (0.20 mL, 2.5 M solution in hexane, 0.5 mmol) was added dropwise over 2 min affording a yellow solution and the mixture was then stirred for 1.5h at -78 °C. The cooling bath was removed, CO_2 was generated from dry ice (~20 g) and bubbled through the reaction mixture via cannula connected to a drying tube packed with $CaCl_2$ for up to 1 h, upon which the reaction solution regained the initial red color. After the reaction had reached rt, the solvent was removed *in vacuo*. On the bench-top open to air, the red-violet solid was dissolved in water (ca. 50 mL), treated with aqueous hydrochloric acid (2.5 mL, 2 N), stirred for 30 min then filtered, washed with water (2 x 30 mL) and dried to give [Fe(coohphtmeimb)$_2$]PF_6. The complex was dissolved in a small amount of methanol and precipitated by addition of diethyl ether. Single crystals (red-violet) were grown from acetonitrile: methanol (3:2) solution with slow diffusion of diethyl ether (0.098 g, Yield: 86%). ^{1}H NMR (400 MHz, DMSO-d_6, 25 mM): δ(ppm) 14.96-14.94 (m, 4H, H_{Ph-c}), 11.00-10.99 (m, 4H, H_{Ph-b}), 5.06 (s, 18H, H_{Im-f}), 0.95 (s, 6H, H_{Im-d}), -12.19 (s, 6H, H_{Im-e}); $^{13}C\{^1H\}$ NMR (100.3 MHz, DMSO-d_6, 25 mM): δ (ppm) 170.1 (C_{Ph-h}), 140.0 (C_{Ph-c}), 133.7 (C_{Ph-b}), 132.9 (C_{Ph-a}), 49.0 (C_{Im-e}), 16.1 (C_{Im-d}), -29.9 (C_{Im-f}); IR (thin layer film) ν (cm^{-1}) 3560, 1685, 1550, 1454, 1417, 1358, 1285, 1250, 1154, 1100, 1089, 1019, 889, 835, 791, 732, 712, 557; MP 350 °C; ESI–

HRMS (m/z): [($C_{38}H_{40}O_4N_{12}B_2Fe$)−($PF_6$)]$^+$ calcd for $C_{38}H_{40}O_4N_{12}B_2Fe$, 806.2831; found, 806.2841; Elemental analysis: (% calculated, % found for $C_{38}H_{44}N_{12}PB_2F_6O_2Fe$): C (47.98, 47.70), H (4.24, 4.32), N (17.67, 17.72).

S2. ^{1}H and ^{13}C{^{1}H} NMR Spectra

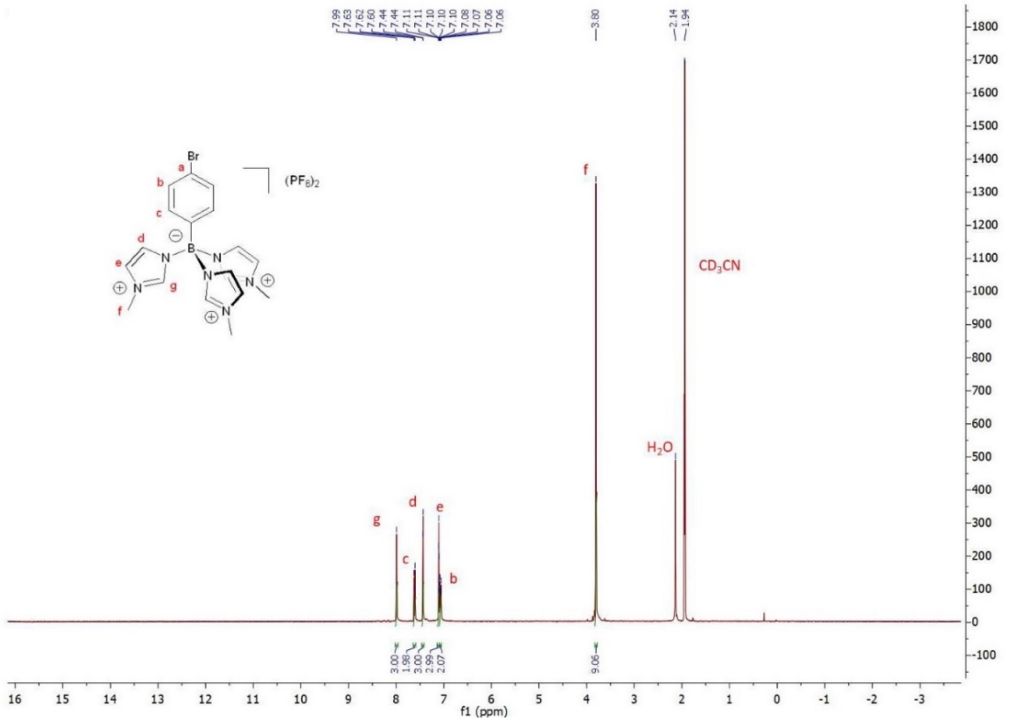

Figure S1. ^{1}H NMR spectrum of ligand [brphtmeimbH_3](PF_6)$_2$ (5 mM) in CD_3CN.

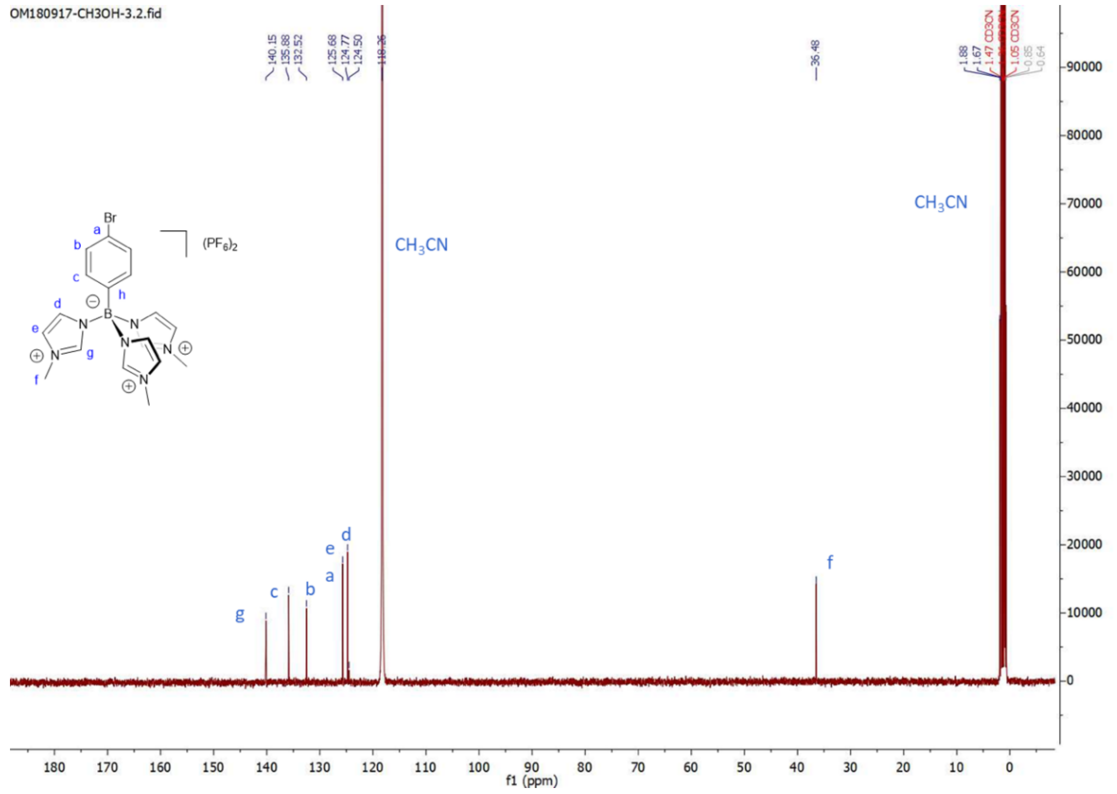

Figure S2. $^{13}C\{^{1}H\}$ NMR spectrum of ligand [brphtmeimbH$_3$](PF$_6$)$_2$ (5 mM) in CD$_3$CN.

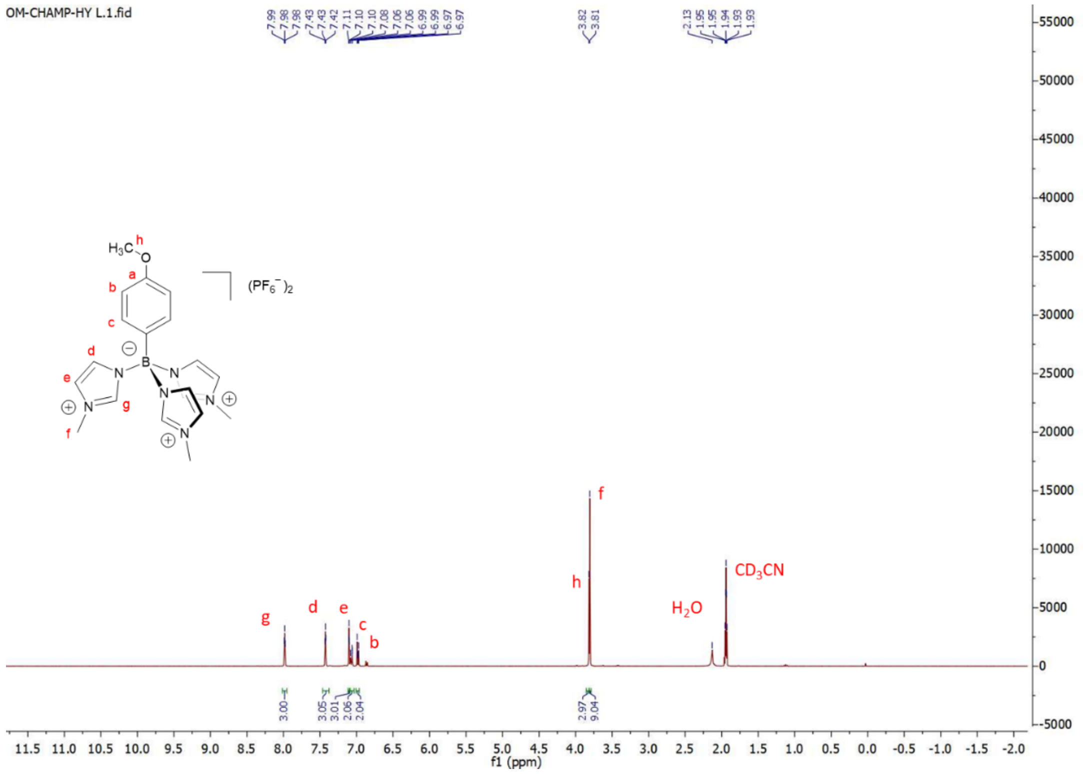

Figure S3. ^{1}H NMR spectrum of ligand [meophtmeimbH_3]$(PF_6)_2$ (5 mM) in CD_3CN.

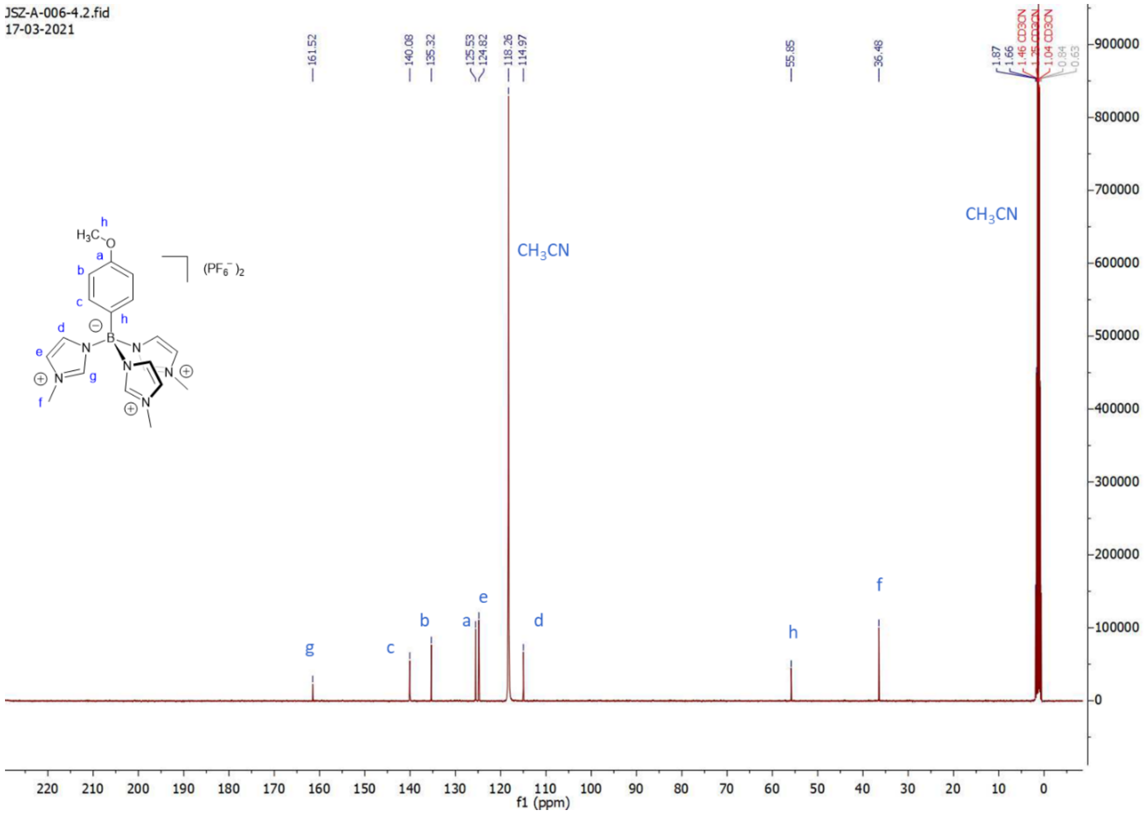

Figure S4. $^{13}C\{^{1}H\}$ NMR spectrum of ligand $[meophtmeimbH_3](PF_6)_2$ (5 mM) in CD_3CN.

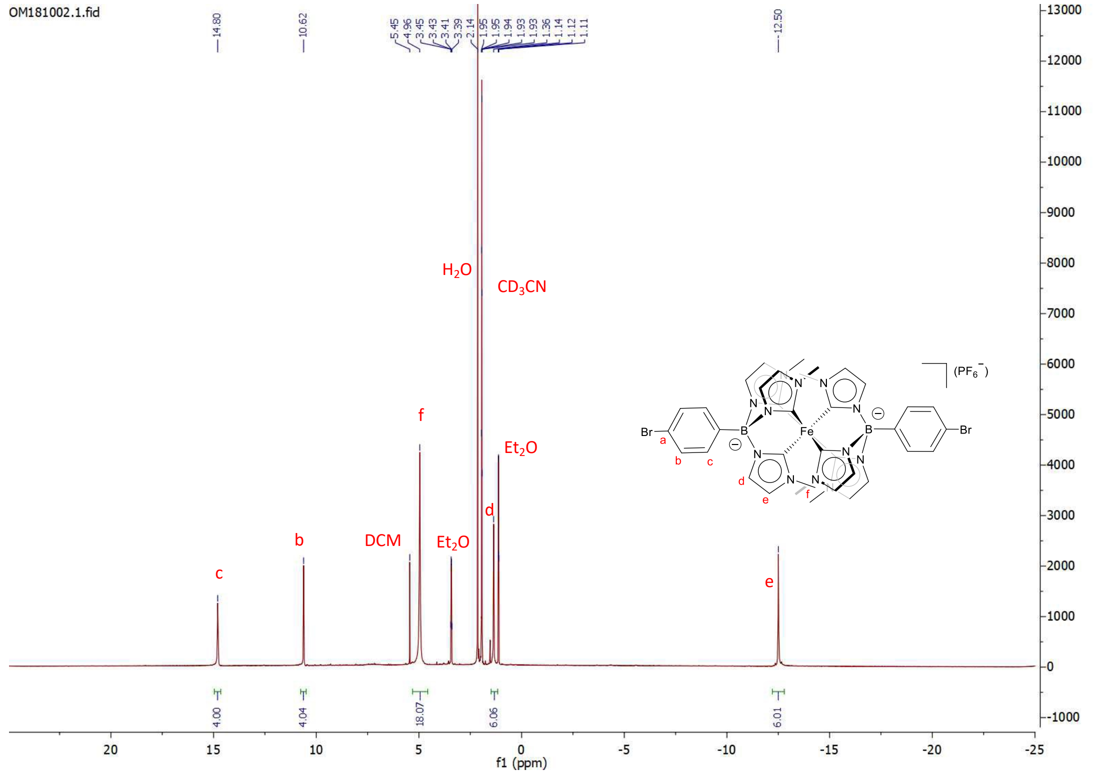

Figure S5. ^{1}H NMR spectrum of complex [Fe(brphtmeimb)$_2$]PF$_6$ (25 mM) in CD$_3$CN.

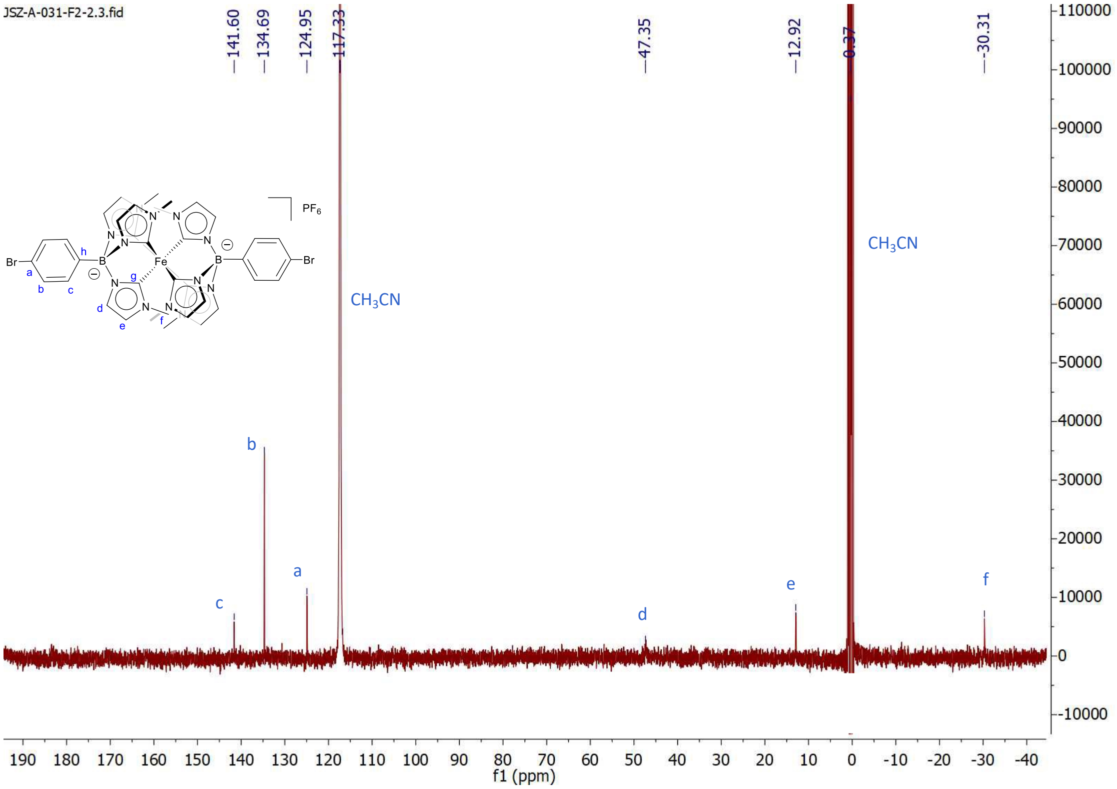

Figure S6. $^{13}C\{^{1}H\}$ NMR spectrum of complex $[Fe(brphtmeimb)_2]PF_6$ (25 mM) in CD_3CN.

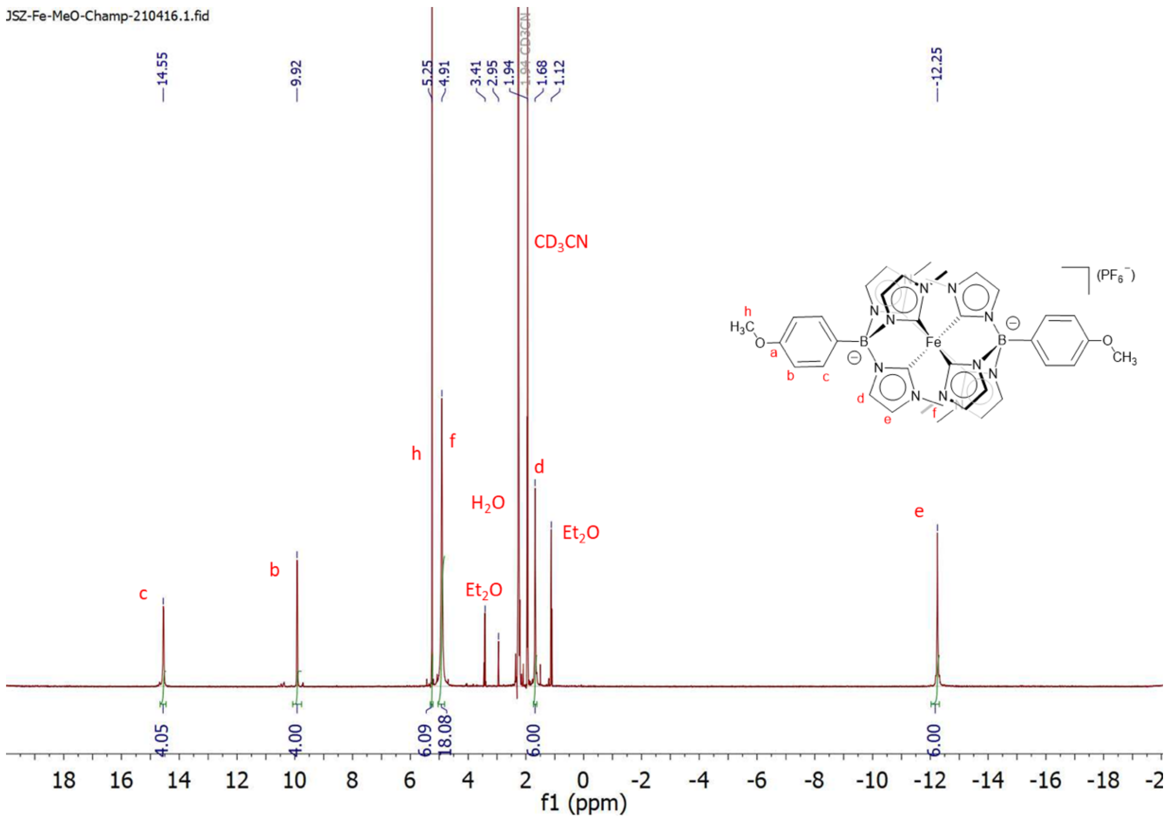

Figure S7. ^{1}H NMR spectrum of complex [Fe(meophtmeimb)$_2$]PF_6 (25 mM) in CD_3CN.

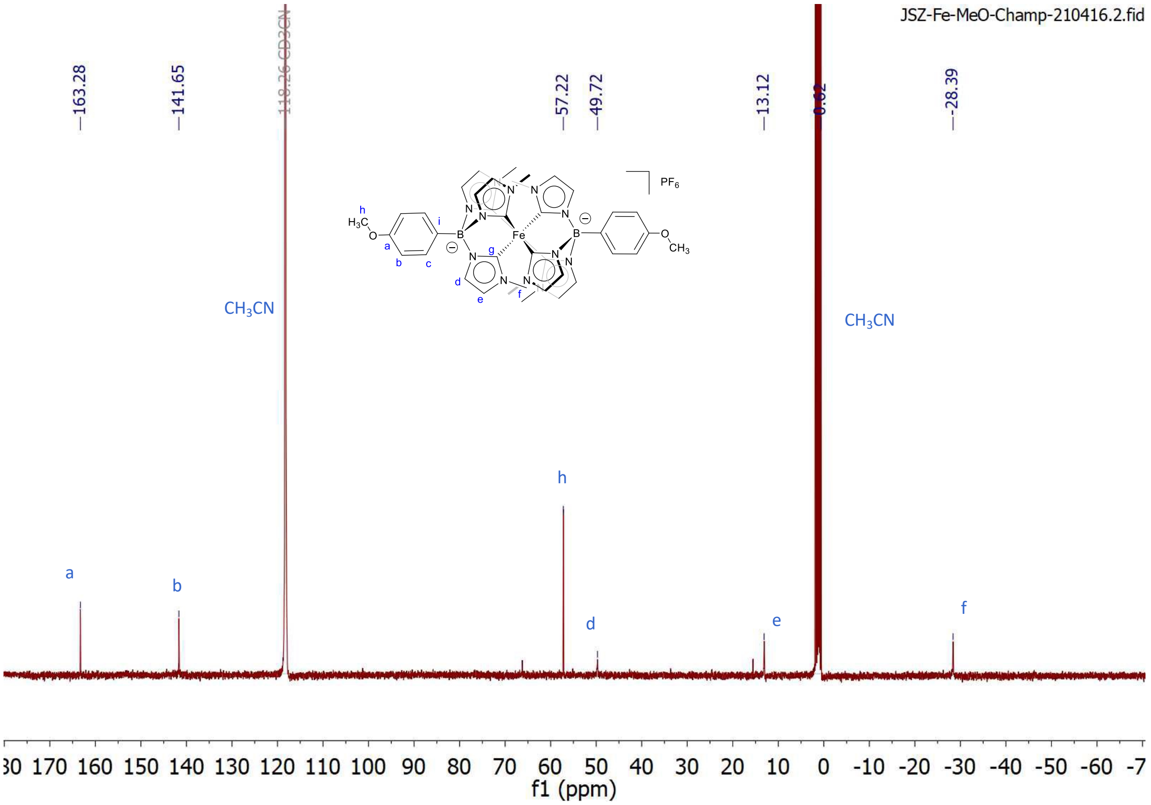

Figure S8. $^{13}C\{^{1}H\}$ NMR spectrum of complex $[Fe(meophtmeimb)_2]PF_6$ (25 mM) in CD_3CN.

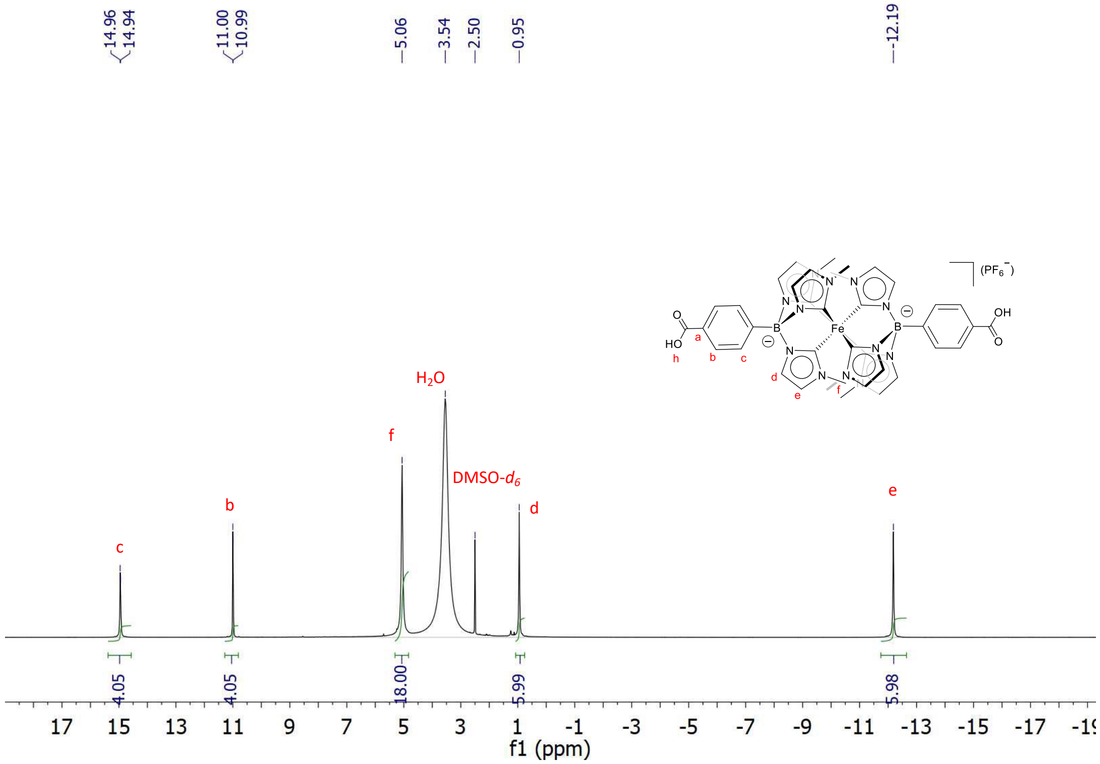

Figure S9. ^{1}H NMR spectrum of complex [Fe(coohphtmeimb)$_2$]PF$_6$ (25 mM) in CD$_3$CN.

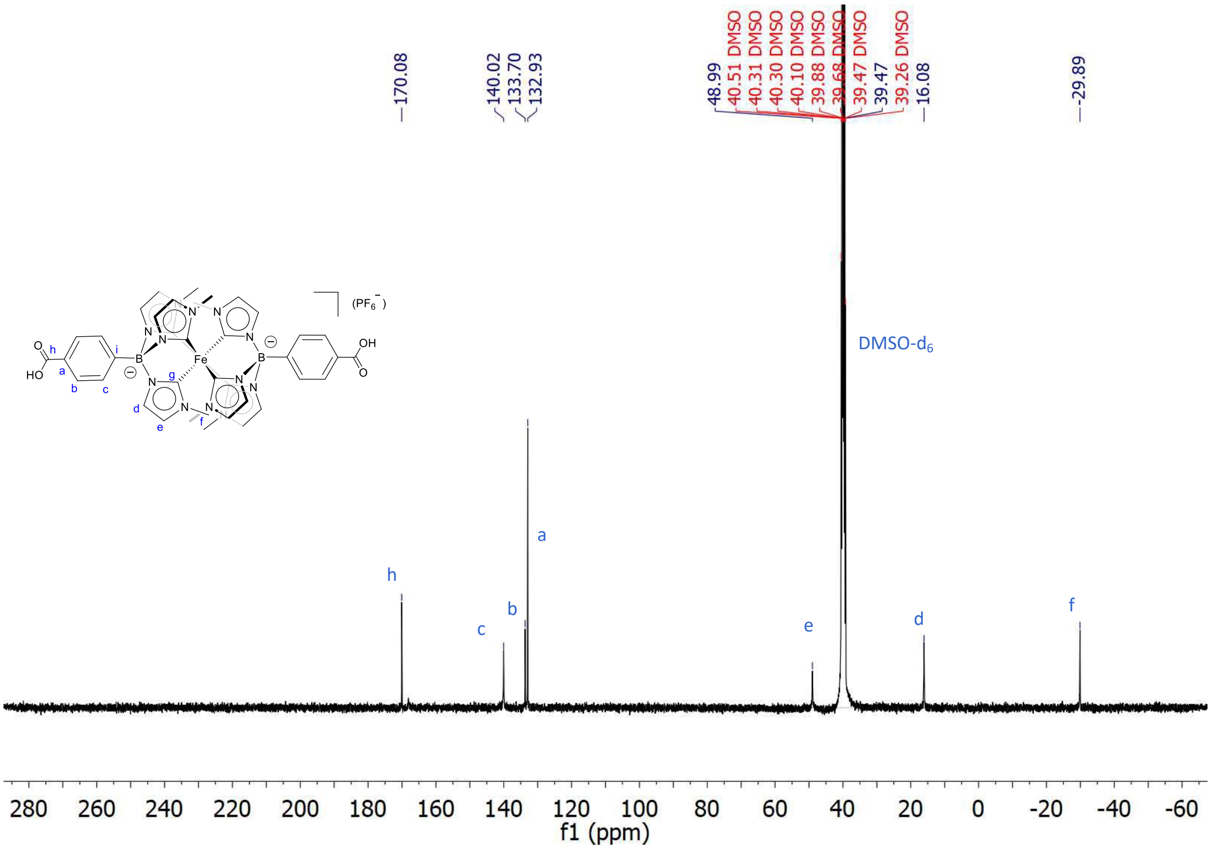

Figure S10. $^{13}C\{^1H\}$ NMR spectrum of complex $[Fe(coohphtmeimb)_2]PF_6$ (25 mM) in CD_3CN.

S3. HR-MS Spectra

Single Mass Analysis
Tolerance = 2.0 mDa / DBE: min = -0.5, max = 100.0
Element prediction: Off
Number of isotope peaks used for i-FIT = 3
Monoisotopic Mass, Odd Electron Ions
37 formula(e) evaluated with 1 results within limits (all results (up to 1000) for each mass)
Elements Used:
C: 34-39 H: 0-100 B: 2-2 N: 10-12 56Fe: 0-1 Br: 0-2

Mass	Calc. Mass	mDa	PPM	DBE	Formula	Fit Conf %	C	H	B	N	56Fe	Br
874.1249	874.1245	0.4	0.5	24.0	C36 H38 B2 N12 56Fe Br2	n/a	36	38	2	12	1	2

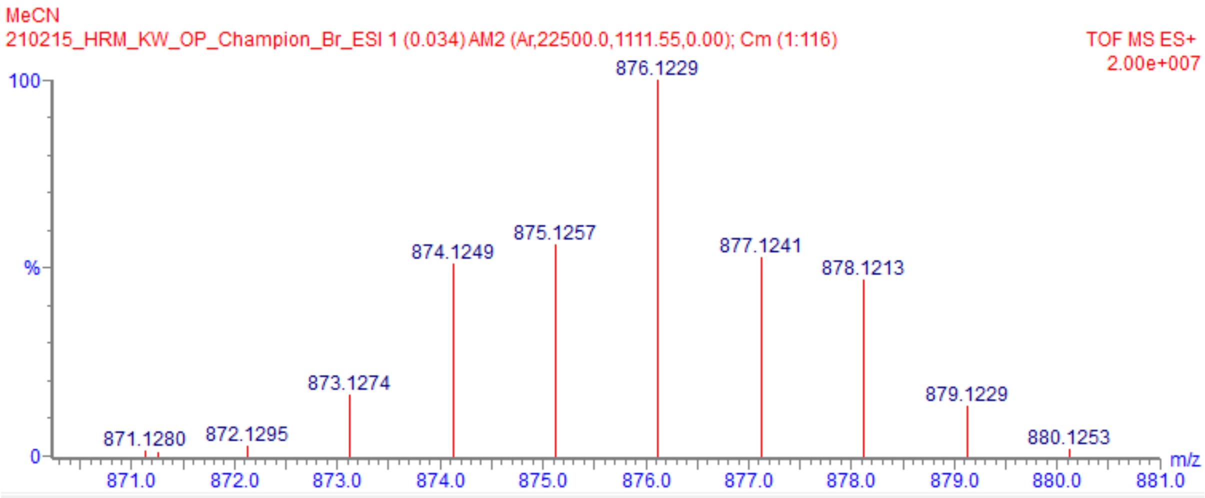

Figure S11. HRMS spectrum of complex $[Fe(brphtmeimb)_2]PF_6$.

Single Mass Analysis
Tolerance = 2.0 mDa / DBE: min = -0.5, max = 100.0
Element prediction: Off
Number of isotope peaks used for i-FIT = 3
Monoisotopic Mass, Odd Electron Ions
29 formula(e) evaluated with 1 results within limits (all results (up to 1000) for each mass)
Elements Used:
C: 34-39 H: 0-100 B: 2-2 N: 10-12 O: 1-2 56Fe: 0-1

Mass	Calc. Mass	mDa	PPM	DBE	Formula	Fit Conf %	C	H	B	N	O	56Fe
778.3265	778.3246	1.9	2.4	24.0	C38 H44 B2 N12 O2 56Fe	n/a	38	44	2	12	2	1

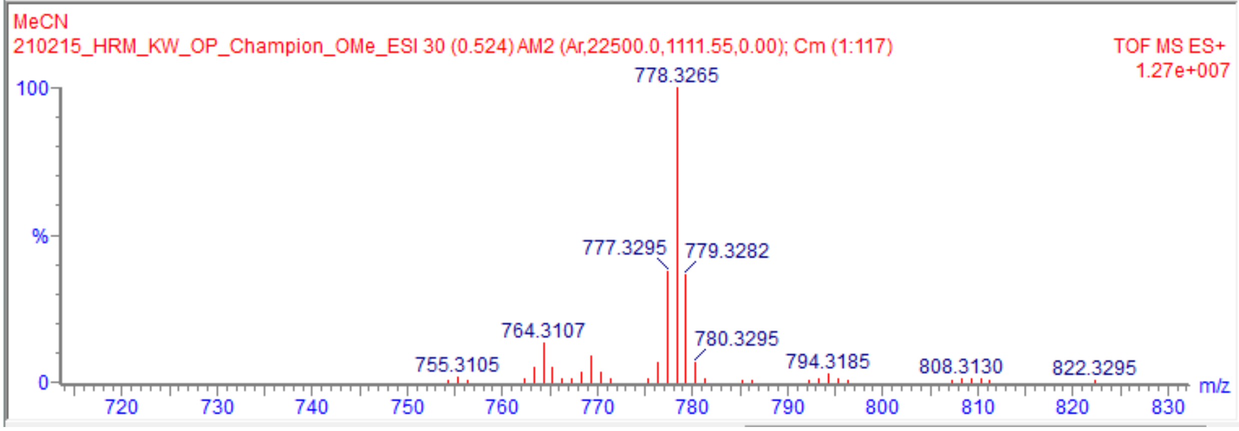

Figure S12. HRMS spectrum of complex $[Fe(brphtmeimb)_2]PF_6$.

Single Mass Analysis
Tolerance = 2.0 mDa / DBE: min = -0.5, max = 100.0
Element prediction: Off
Number of isotope peaks used for i-FIT = 3
Monoisotopic Mass, Odd Electron Ions
324 formula(e) evaluated with 3 results within limits (all results (up to 1000) for each mass)
Elements Used:
C: 0-50 H: 0-70 B: 0-2 N: 11-13 O: 3-5 Fe: 0-1

Mass	Calc. Mass	mDa	PPM	DBE	Formula	i.	Fit Conf %	C	H	B	N	O	Fe
806.2844	806.2852	-0.8	-1.0	27.0	C41 H42 N12 O3 Fe		72...0.00	41	42		12	3	1
	806.2831	1.3	1.6	26.0	C38 H40 B2 N12 O4 Fe		16...0.00	38	40	2	12	4	1
	806.2826	1.8	2.2	35.0	C45 H34 N12 O4		40...100.00	45	34		12	4	

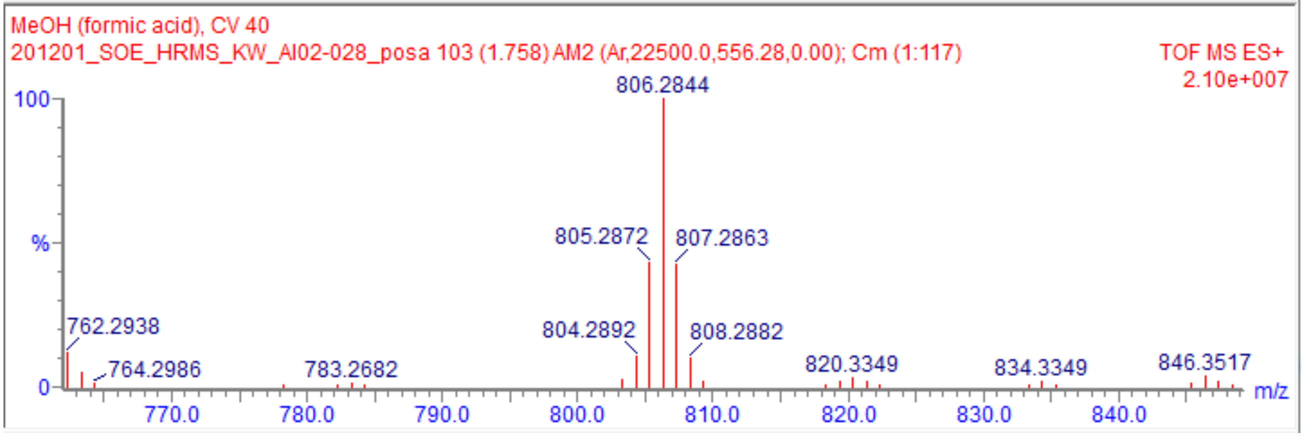

Figure S13. HRMS spectrum of complex $[Fe(brphtmeimb)_2]PF_6$.

S4. Single crystal X-ray diffraction

All SC-XRD measurements were performed using graphite-monochromatized Mo Kα radiation (λ = 0.71073 Å) using the Agilent Xcalibur Sapphire3 diffractometer high-brilliance IμS radiation source. Data collections were performed at 110 K for $[Fe(brphtmeimb)_2]PF_6$ and 293 K for $[Fe(meophtmeimb)_2]PF_6$. The structures were solved by direct methods and refined by full-matrix least-squares techniques against F^2 using all data (SHELXT, SHELXS).[1] All non-hydrogen atoms were refined with anisotropic displacement parameters if not stated otherwise. The OLEX2 solvent masking was used to treat diffuse scattering in $[Fe(mophtmeimb)_2]PF_6$ using OLEX2 software.[2] Non-hydrogen atoms were refined anisotropically.

Table S1. Crystal data and structure refinement for $[Fe(brphtmeimb)_2]PF_6$.

Identification code	$[Fe(brphtmeimb)_2]PF_6$	
Empirical formula	$C_{36}H_{38}B_2Br_2F_6FeN_{12}P$	
Formula weight	1021.04	
Temperature	110(2) K	
Wavelength	0.71073 Å	
Crystal system	Triclinic	
Space group	P-1	
Unit cell dimensions	a = 11.2793(6) Å	α= 91.126(4)°.
	b = 12.6429(6) Å	β= 103.679(4)°.
	c = 17.3260(7) Å	γ = 108.757(5)°.
Volume	2260.8(2) $Å^3$	
Z	2	
Density (calculated)	1.500 Mg/m^3	
Absorption coefficient	2.202 mm^{-1}	
F(000)	1026	
Crystal size	0.2 x 0.2 x 0.1 mm^3	
Theta range for data collection	3.327 to 29.014°.	
Index ranges	-13<=h<=15, -17<=k<=15, -17<=l<=22	
Reflections collected	20831	
Independent reflections	10186 [R(int) = 0.0445]	
Completeness to theta = 25.000°	99.8 %	
Absorption correction	Semi-empirical from equivalents	
Max. and min. transmission	1.00000 and 0.75742	
Refinement method	Full-matrix least-squares on F^2	
Data / restraints / parameters	10186 / 0 / 550	
Goodness-of-fit on F^2	1.022	
Final R indices [I>2sigma(I)]	R1 = 0.0583, wR2 = 0.1253	
R indices (all data)	R1 = 0.0907, wR2 = 0.1403	
Extinction coefficient	n/a	
Largest diff. peak and hole	1.124 and -0.687 e.$Å^{-3}$	

Table S2. Crystal data and structure refinement for $[Fe(meophtmeimb)_2]PF_6$

Identification code	$[Fe(meophtmeimb)_2]PF_6$	
Empirical formula	$C_{38}H_{44}B_2F_6FeN_{12}O_2P$	
Formula weight	923.29	
Temperature	293(2) K	
Wavelength	0.71073 Å	
Crystal system	Orthorhombic	
Space group	Pcca	
Unit cell dimensions	a = 25.004(2) Å	α= 90°.
	b = 10.1122(8) Å	β= 90°.
	c = 19.6779(18) Å	γ = 90°.
Volume	4975.4(8) $Å^3$	
Z	4	
Density (calculated)	1.233 Mg/m^3	
Absorption coefficient	0.401 mm^{-1}	
F(000)	1908	
Crystal size	0.100 x 0.100 x 0.100 mm^3	
Theta range for data collection	3.317 to 29.201°.	
Index ranges	-29<=h<=33, -11<=k<=12, -25<=l<=25	
Reflections collected	19332	
Independent reflections	5711 [R(int) = 0.1138]	
Completeness to theta = 25.000°	99.7 %	
Absorption correction	Semi-empirical from equivalents	
Max. and min. transmission	1.00000 and 0.94447	
Refinement method	Full-matrix least-squares on F^2	
Data / restraints / parameters	5711 / 0 / 286	
Goodness-of-fit on F^2	0.961	
Final R indices [I>2sigma(I)]	R1 = 0.0906, wR2 = 0.1927	
R indices (all data)	R1 = 0.2057, wR2 = 0.2503	
Extinction coefficient	n/a	
Largest diff. peak and hole	0.607 and -0.458 e.$Å^{-3}$	

Table S3. Crystal data and structure refinement for [Fe(coohphtmeimb)$_2$]PF_6.

Identification code	[Fe(coohphtmeimb)$_2$]PF_6	
Empirical formula	$C_{38}H_{38}B_2F_6FeN_{12}O_4P$	
Formula weight	949.24	
Temperature	293(2) K	
Wavelength	0.71073 Å	
Crystal system	Monoclinic	
Space group	I2/m	
Unit cell dimensions	a = 8.7439(3) Å	α= 90°.
	b = 13.5391(4) Å	β= 91.301(3)°.
	c = 20.3819(7) Å	γ = 90°.
Volume	2412.28(14) $Å^3$	
Z	2	
Density (calculated)	1.307 Mg/m^3	
Absorption coefficient	0.419 mm^{-1}	
F(000)	974	
Crystal size	0.2 x 0.1 x 0.1 mm^3	
Theta range for data collection	3.009 to 29.303°.	
Index ranges	-10<=h<=11, -17<=k<=14, -26<=l<=25	
Reflections collected	9720	
Independent reflections	2987 [R(int) = 0.0313]	
Completeness to theta = 25.000°	99.6 %	
Absorption correction	Semi-empirical from equivalents	
Max. and min. transmission	1.00000 and 0.98992	
Refinement method	Full-matrix least-squares on F^2	
Data / restraints / parameters	2987 / 30 / 184	
Goodness-of-fit on F^2	1.137	
Final R indices [I>2sigma(I)]	R1 = 0.0789, wR2 = 0.2270	
R indices (all data)	R1 = 0.0871, wR2 = 0.2332	
Extinction coefficient	n/a	
Largest diff. peak and hole	0.598 and -0.629 e.$Å^{-3}$	

Table S4. Selected (Fe-C) bond lengths and (C-Fe-C) bond angels in comparison with the parent complex [Fe(phtmeimb)$_2$]PF_6

Complex	Fe–C (Å)[a]	C–Fe–C (°)
[Fe(phtmeimb)$_2$]$^{+}$,[23]	Fe(1)-C(17): 2.009(4)	C(17)-Fe(1)-C(9): 86.42(18)
	Fe(1)-C(9): 2.003(4)	C(17)-Fe(1)-C(13): 87.48(17)
	Fe(1)-C(13): 1.984(5)	C(9)-Fe(1)-C(13): 87.12(19)
[Fe(brphtmeimb)$_2$]$^{+}$	Fe(1)-C(4): 1.964(4)	C(4)-Fe(1)-C(5): 87.10(15)
	Fe(1)-C(5): 1.975(4)	C(4)-Fe(1)-C(9): 87.36(16)
	Fe(1)-C(9): 2.001(4)	C(5)-Fe(1)-C(9): 87.30(15)
[Fe(meophtmeimb)$_2$]$^{+}$	C(15)-Fe(1): 1.977(5)	C(19)-Fe(1)-C(15): 86.70(2)
	C(19)-Fe(1): 1.965(5)	C(19)-Fe(1)-C(11): 87.10(2)
	C(11)-Fe(1): 1.986(5)	C(15)-Fe(1)-C(11): 87.10(2)
[Fe(coohphtmeimb)$_2$]$^{+}$	Fe(1)-C(13): 1.977(5)	C(13)-Fe(1)-C(9): 87.27(13)
	Fe(1)-C(9): 1.988(3)	

[a]For the numbering of the atoms, see Fig. 2.

Table S5. Selected bond lengths (Å) and bond angles (°) of all the complexes.

Compound	Bond lengths (Å)	Bond angles (°)
[Fe(brphtmeimb)$_2$]PF$_6$	Fe(1)-C(4): 1.964(4)<br>Fe(1)-C(5): 1.975(4)<br>Fe(1)-C(9): 2.001(4)<br>Fe(2)-C(19): 1.992(4)<br>Fe(2)-C(23): 1.961(4)<br>Fe(2)-C(27): 1.991(4)	C(4)-Fe(1)-C(5): 87.10(15)<br>C(4)-Fe(1)-C(9): 87.36(16)<br>C(5)-Fe(1)-C(9): 87.30(15)<br>N(5)-C(9)-Fe(1): 121.6(3)<br>N(6)-C(9)-Fe(1): 133.0(3)<br>C(23)-Fe(2)-C(19): 87.24(16)<br>C(23)-Fe(2)-C(27): 87.00(15)<br>C(27)-Fe(2)-C(19): 87.67(17)<br>N(7)-C(19)-Fe(2): 121.4(3)<br>N(8)-C(19)-Fe(2): 133.6(3)<br>N(9)-C(23)-Fe(2): 120.7(3)<br>N(9)-C(23)-N(10): 105.3(3)
[Fe(meophtmeimb)$_2$]PF$_6$	C(15)-Fe(1): 1.977(5)<br>C(19)-Fe(1): 1.965(5)<br>C(11)-Fe(1): 1.986(5)	C(19)-Fe(1)-C(15): 86.7(2)<br>C(19)-Fe(1)-C(11): 87.1(2)<br>C(15)-Fe(1)-C(11): 87.1(2)<br>N(2)-C(11)-Fe(1): 122.0(4)<br>N(1)-C(11)-Fe(1): 132.0(4)<br>N(4)-C(15)-Fe(1): 121.8(4)<br>N(3)-C(15)-Fe(1): 133.3(4)

		N(5)-C(19)-Fe(1): 120.4(4)<br>N(6)-C(19)-Fe(1): 133.0(4)
$[Fe(coohphtmeimb)_2]PF_6$	Fe(1)-C(9)#1: 1.988(3)<br>Fe(1)-C(9): 1.988(3)<br>Fe(1)-C(9)#2: 1.988(3)<br>Fe(1)-C(9)#3: 1.988(3)<br>Fe(1)-C(13): 1.977(5)<br>Fe(1)-C(13)#3: 1.977(5)	C(9)#1-Fe(1)-C(9)#2: 180.00(14)<br>C(9)#1-Fe(1)-C(9): 86.61(19)<br>C(9)#3-Fe(1)-C(9): 180.00(14)<br>C(9)#1-Fe(1)-C(9)#3: 93.39(19)<br>C(9)#2-Fe(1)-C(9)#3: 86.61(19)<br>C(9)#2-Fe(1)-C(9): 93.39(19)<br>C(13)-Fe(1)-C(9)#3: 92.73(13)<br>C(13)-Fe(1)-C(9)#2: 92.73(13)<br>C(13)#3-Fe(1)-C(9)#3: 87.27(13)<br>C(13)-Fe(1)-C(9): 87.27(13)<br>C(13)#3-Fe(1)-C(9): 92.73(13)<br>C(13)-Fe(1)-C(9)#1: 87.27(13)<br>C(13)#3-Fe(1)-C(9)#1: 92.73(13)<br>C(13)#3-Fe(1)-C(9)#2: 87.27(13)<br>C(13)#3-Fe(1)-C(13): 180.0

S5. Magnetic susceptibility and magnetization measurements

The magnetic data were acquired on a Quantum-Design MPMS-XL SQUID magnetometer. Susceptibility data were acquired in a static field of 1.0 KOe. Magnetization data were obtained with selected fields from $B = 1$ to 50 KOe in the temperature range $T = 2$-10 K. The polycrystalline samples were measured on a compacted powder sample in a polycarbonate capsule. Data were corrected empirically for TIP and the diamagnetic contribution to the sample moment from the sample holder and sample was corrected through background measurements and Pascal constants, respectively.

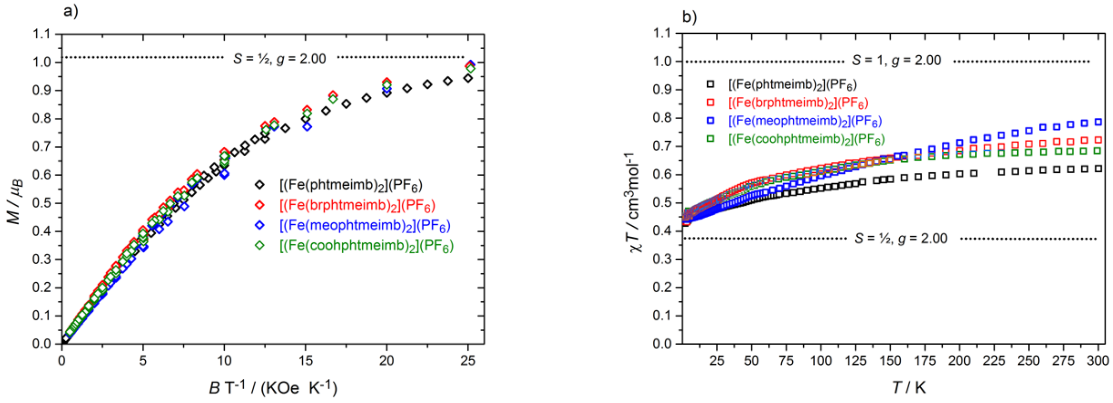

Figure S14. Experimental magnetic data and theoretical limiting values for [Fe(phtmeimb)$_2$]PF$_6$,[23] (black), [Fe(brphtmeimb)$_2$]PF$_6$ (red), [Fe(meophtmeimb)$_2$]PF$_6$ (blue) and [Fe(coohphtmeimb)$_2$]PF$_6$ (green). a) Magnetization data versus B/T recorded at fields of 0.1-5 T and temperatures 2-10 K. b) Magnetic susceptibility versus temperature.

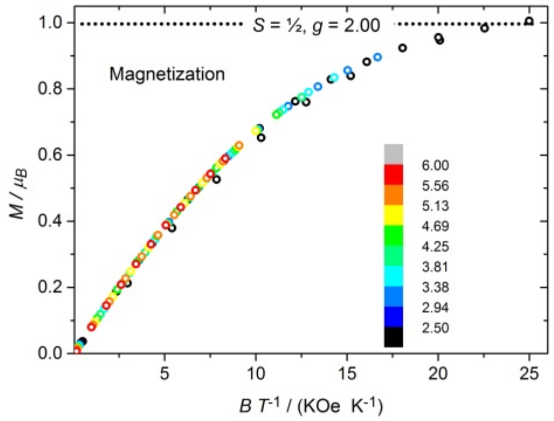

Figure S15. Magnetization data of [Fe(brphtmeimb)$_2$]PF_6 recorded at fields 1 to 50 KOe and temperatures 2-10K. The insert color coding identifies the temperature. The superimposable curves for all fields is expected for an *S*=½ spin-system.

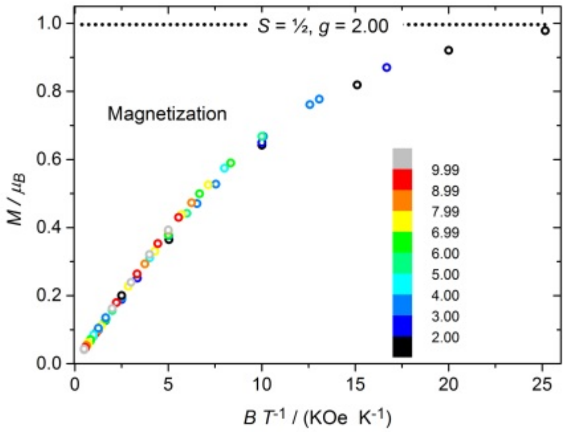

Figure S16. Magnetization data of [Fe(meophtmeimb)$_2$]PF_6 recorded at fields 1 to 50 KOe and temperatures 2-10K. The insert color coding identifies the temperature. The superimposable curves for all fields is expected for an *S*=½ spin-system.

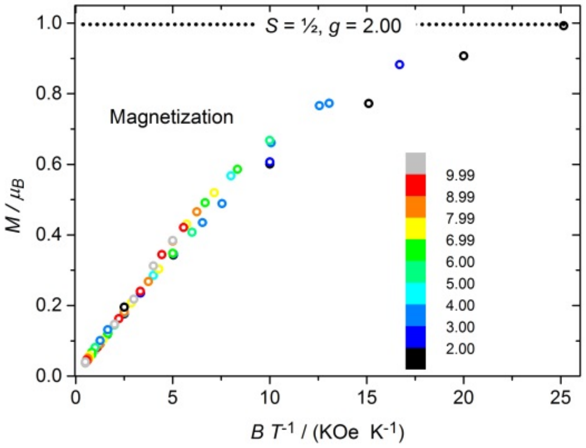

Figure S17. Magnetization data of [Fe(coohphtmeimb)$_2$]PF$_6$ recorded at fields 1 to 50 KOe and temperatures 2-10K. The insert color coding identifies the temperature. The superimposable curves for all fields is expected for an S=½ spin-system.

S6. Electron paramagnetic resonance measurements

X-band EPR measurements were performed on a BrukerELEXYS E500 spectrometer equipped with a SuperX EPR049 microwave bridge and a cylindrical TE011 ER 4122SHQE cavity. The temperature was controlled using an Oxford Instruments ESR 900 flow cryostat. EPR spectra were analyzed using the XEPR software package. Helium temperature EPR spectra were recorded on a Bruker E580-ELEXSYS spectrometer equipped with an Oxford 900 liquid helium cryostat and an ITC 503 temperature controller. An ER4116DM dual mode X-band resonator of rectangular type (TE102 for perpendicular and TE012 for parallel mode) was used for measurements. Modulation frequency of 100 kHz was applied for all spectral recordings. Samples of $[Fe(brphtmeimb)_2]PF_6$, $[Fe(meophtmeimb)_2]PF_6$, and $[Fe(coohphtmeimb)_2]PF_6$ (ca. 1 mM) were dissolved in argon purged, dry acetonitrile and transferred into EPR tubes under dim-light. Samples in different oxidation states were obtained by exhaustive controlled potential electrolysis at -1.48 V, -0.78 V, and +0.62 V vs. fc that yield the Fe(II), Fe(III) and Fe(IV) state, respectively. Samples of 150 μl were transferred from the electrolysis cell to argon filled EPR tubes using an argon flushed gas tight syringe. All samples were stored in liquid nitrogen and in darkness before EPR examination. None of $[Fe(brphtmeimb)_2]PF_6$, $[Fe(meophtmeimb)_2]PF_6$, and $[Fe(coohphtmeimb)_2]$ or any of the electrolyzed samples showed any EPR signal in perpendicular mode, irrespective of temperature (4 to 25 K) and microwave power (e.g. 0.2 mW and 0.8 mW) as similar to the parent $[Fe(phtmeimb)_2]PF_6$ complex. Also, in parallel mode at liquid helium temperature all samples were EPR silent. Equally, no EPR signal from $[Fe(brphtmeimb)_2]PF_6$, $[Fe(meophtmeimb)_2]PF_6$, and $[Fe(coohphtmeimb)_2]PF_6$ was found in butyronitrile solution or a powder sample (dispersed in mineral oil).

The absence of any EPR signal clearly distinguishes [Fe(brphtmeimb)$_2$]PF$_6$, [Fe(meophtmeimb)$_2$]PF$_6$, and [Fe(coohphtmeimb)$_2$]PF$_6$ from the Fe(III) state of similar NHC complexes previously synthesized in our laboratory that showed EPR signals with axial anisotropic character, typical for low spin (S=1/2) Fe(III).

S7. Mößbauer spectroscopy

Mössbauer measurements were carried out in an Oxford Instrument flow cryostat at 80 K and at 295 K using a ^{57}CoRh source held at room temperature. The studied powder materials were mixed with inert BN, pressed, and formed as pastille absorbers with a concentration of about 40 mg/cm^2 of studied substances. Calibration spectra were recorded from a natural iron metal foil held at 295 K. The resulting spectra were analysed using a least square Mössbauer fitting program.

The electric quadrupole splittings |QS| and center shifts CS at 80K, versus natural Fe at room temperature, of the [Fe(brphtmeimb)$_2$]PF$_6$, [Fe(meophtmeimb)$_2$]PF$_6$, and [Fe(coohphtmeimb)$_2$]PF$_6$ were compared with previously reported Fe-NHC complexes and shown in Figure S7.1 and S7.2.

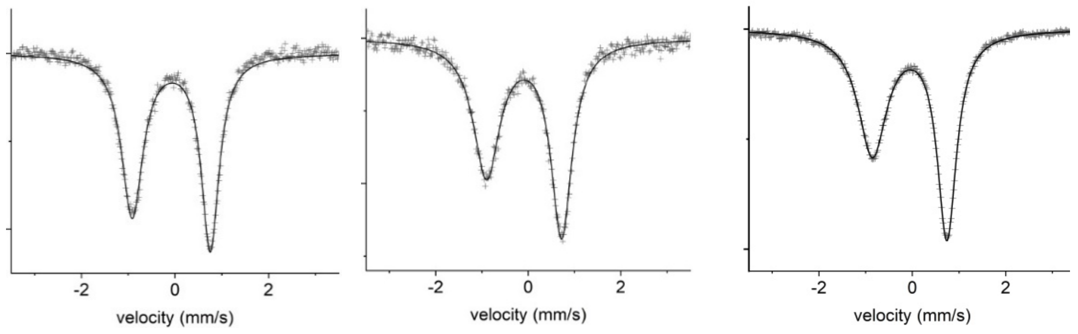

[Fe(brphtmeimb)$_2$]PF$_6$ [Fe(meophtmeimb)$_2$]PF$_6$ [Fe(coohphtmeimb)$_2$]PF$_6$

Figure S18. Low temperature (80 K) ^{57}Fe Mößbauer spectra of studied complexes. The absorptions are about 1% of the background values.

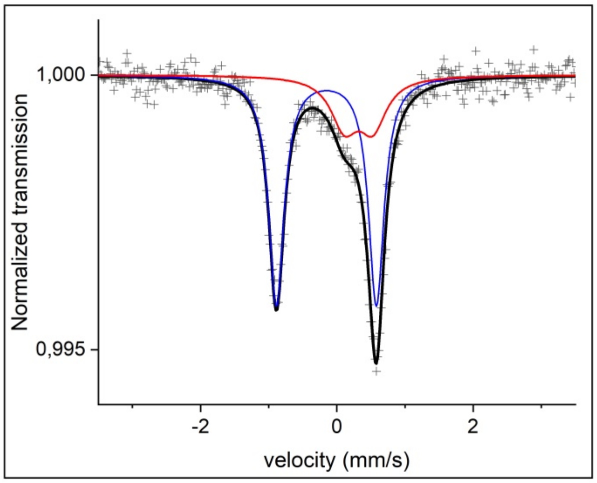

Figure S19. Mössbauer spectrum of [Fe(meophtmeimb)$_2$]PF_6 at 295 K. The blue and red patterns emanates from Fe(III) and Fe(II) valence states, respectively. The fitted center shifts CS are -0.15(1) mm/s and 0.32(4) mm/s versus natural Fe at 295 K and the electric quadrupole splittings |QS| are 1.47(1) mm/s and 0.42(6) mm/s, respectively. The relative intensity of the Fe(II) component is 25(4) % at 295 K. Possible Fe(II) contaminations could include Fe(II)Cl_2 or $Fe(OH)_2$, the latter due to the very basic conditions for forming the carbene that will react with Fe(II)Cl_2 to make the (Fe(II)(meophtmeimb)]PF_6 complex. We tried the elemental analysis (EA) using a composition of 3% of Fe(II)Cl_2 and 97% of (Fe(III)(meophtmeimb)]PF_6, and 3% of Fe(II)$(OH)_2$ and 97% of (Fe(III)(meophtmeimb)]PF_6, respectively, And in the first case, the EA ended up within ±0.4% of CHN and in the second case within ± 0.4 for N and H but ±0.6%. Since the NMR spectra of Fe(III)(meophtmeimb)]PF_6 is clean showing only on species, there is no other coordination compound present that could influence the photophysics in the visible region of the spectra, where the excitations in our investigations are taking place. Therefore, the minor contamination of supposably Fe(II)Cl_2 and $Fe(OH)_2$ which have absorption in the far-UV, will not influence our photophysical measurements. With the accuracy reported of the different extinction coefficients given in Table 2, we see no reason to correct for the presence of this minor amount of

impurity(ies) in the samples used when performing the UV/Vis spectroscopy for Fe(III)(meophtmeimb)]PF_6.

Table S6. Results of the fitting procedure of the 80 K Mößbauer spectra. CS is the center shift relative natural Fe held at 295 K, QS is the magnitude of the electric quadrupole splitting, Γ_+ is the Lorentzian line width for the high velocity peak and Γ_- / Γ_+ is the ratio between the low and high velocity peak, respectively.

Complex	CS mm/s	QS mm/s	Γ_+ mm/s	Γ_- / Γ_+
$[Fe(phtmeimb)_2]^+$,[23]	-0.090(5)	1.539(5)	0.536(5)	1.42(5)
$[Fe(brphtmeimb)_2]^+$	-0.081(5)	1.666(5)	0.458(10)	1.21(5)
$[Fe(meophtmeimb)_2]^+$	-0.089(5)	1.620(5)	0.526(5)	1.44(5)
$[Fe(coohphtmeimb)_2]^+$	-0.056(5)	1.595(5)	0.500(6)	1.50(4)

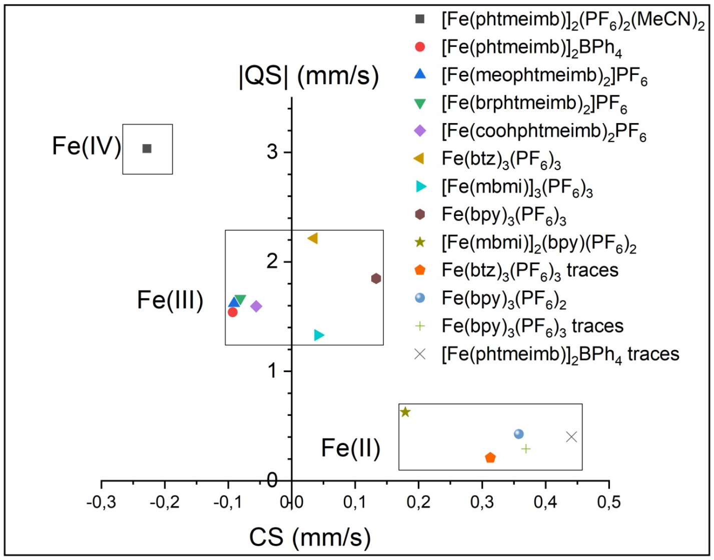

Figure S20. Electric quadrupole splittings |QS| and center shifts CS at 80K, versus natural Fe at room temperature, for $[Fe(brphtmeimb)_2]PF_6$, $[Fe(meophtmeimb)_2]PF_6$, and $[Fe(coohphtmeimb)_2]PF_6$ and previously reported similar complexes.[3,4]

S8. Steady State Spectroscopy

S8.1 Steady State Absorption Spectroscopy

Steady state absorption was measured using the Perkin Elmer Lambda 1050 UV/Vis Spectrophotometer, filling a sample in a quartz-glass cuvette with 1 mm optical path-length (Hellma – QS Glass). For all complexes dry acetonitrile was used as the solvent, except for [Fe(coohphtmeimb)$_2$]PF$_6$ that was measured in dry methanol for solubility reasons (both solvents collected from a solvent dispenser PS-micro, Innovative technology). For reference the same cuvette with only solvent was measured before sample measurement. To establish the extinction coefficient, a dilution series of each complex starting from a stock solution with known concentration was measured. The absorbance at each wavelength was fitted by a linear trend line as a function of concentration, from which the extinction coefficient was calculated.

S8.2 Steady State Emission Spectroscopy

Fresh sample solutions of [Fe(phtmeimb)$_2$]PF$_6$, [Fe(brphtmeimb)$_2$]PF$_6$, [Fe(meophtmeimb)$_2$]PF$_6$ and [Fe(coohphtmeimb)$_2$]PF$_6$ (concentration ~0.3 mM of all) were prepared in acetonitrile (from Sigma-Aldrich Sure/Seal Bottle) and filtered using 0.45/0.20 μm PTFE-filter. The low concentration needed for emission measurements is far below the solubility limit of [Fe(coohphtmeimb)$_2$]PF$_6$ in acetonitrile and thus allowed for usage of the same solvent for all four complexes. Prior to emission measurements the sample quality was checked by steady-state absorption and no significant differences from the known absorption spectra were found. Emission measurements were performed on a Horiba Fluorolog spectrofluorometer in the front face geometry, using a quartz cuvette with 1 cm or 1 mm optical path length (Hellma – QS-glass). To suppress stray light from sample excitation, long-pass filters were inserted for some measurements

at the entrance slit of the detection monochromator. For luminescence excitation measurements a 565 nm long-pass filter (similar to Schott RG565) was used. To estimate the emission quantum yield relative to $[Fe(phtmeimb)_2]PF_6$ subsequent measurements were performed using the same parameters and only changing the sample. To account for differences in sample concentration and extinction coefficient, the recorded emission intensity was scaled by the number of absorbed photons of each sample. Then scaled intensities were compared to calculate the quantum yield.

In Figures S21-S23 the luminescence excitation spectra of $[Fe(brphtmeimb)_2]PF_6$, $[Fe(meophtmeimb)_2]PF_6$ and $[Fe(coohphtmeimb)_2]PF_6$ are plotted together with the absorption spectra of each sample. The agreement between the excitation spectrum and the absorbance suggests that the measured emission indeed comes from the ^{2}LMCT excited state of each sample.

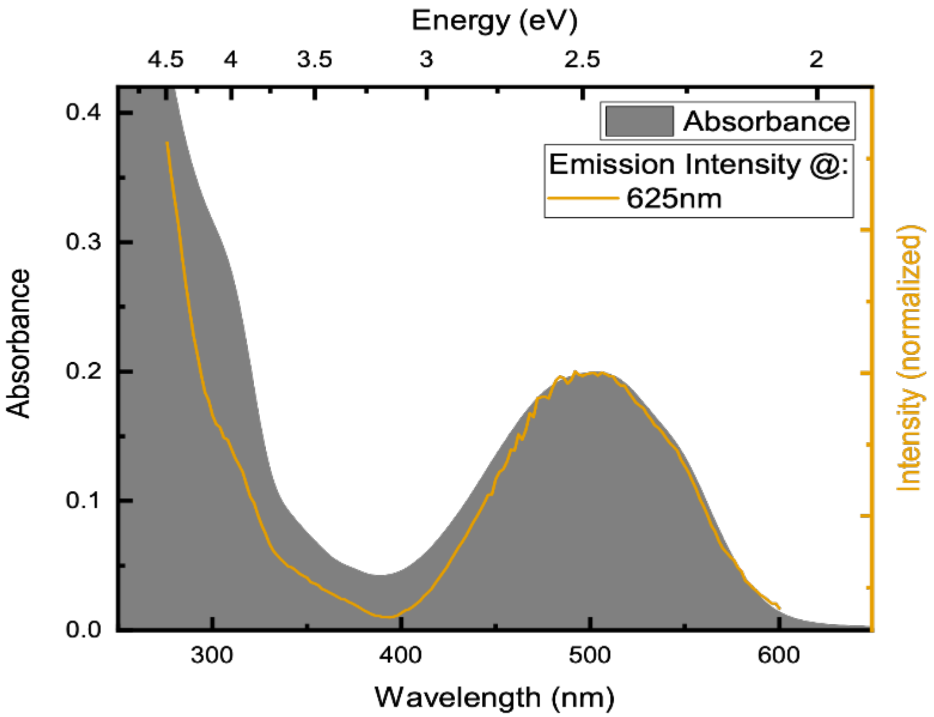

Figure S21. Absorption of $[Fe(brphtmeimb)_2]PF_6$ in acetonitrile (grey area) and luminescence excitation spectrum of the same sample probed at 625 nm.

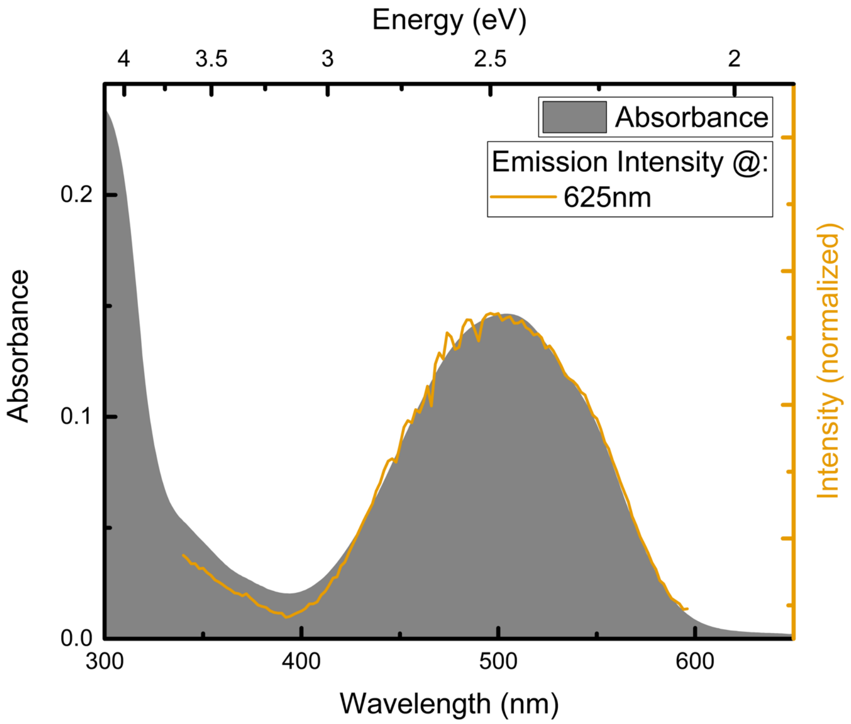

Figure S22. Absorption of [Fe(meophtmeimb)$_2$]PF_6 in acetonitrile (grey area) and luminescence excitation spectrum of the same sample probed at 625 nm.

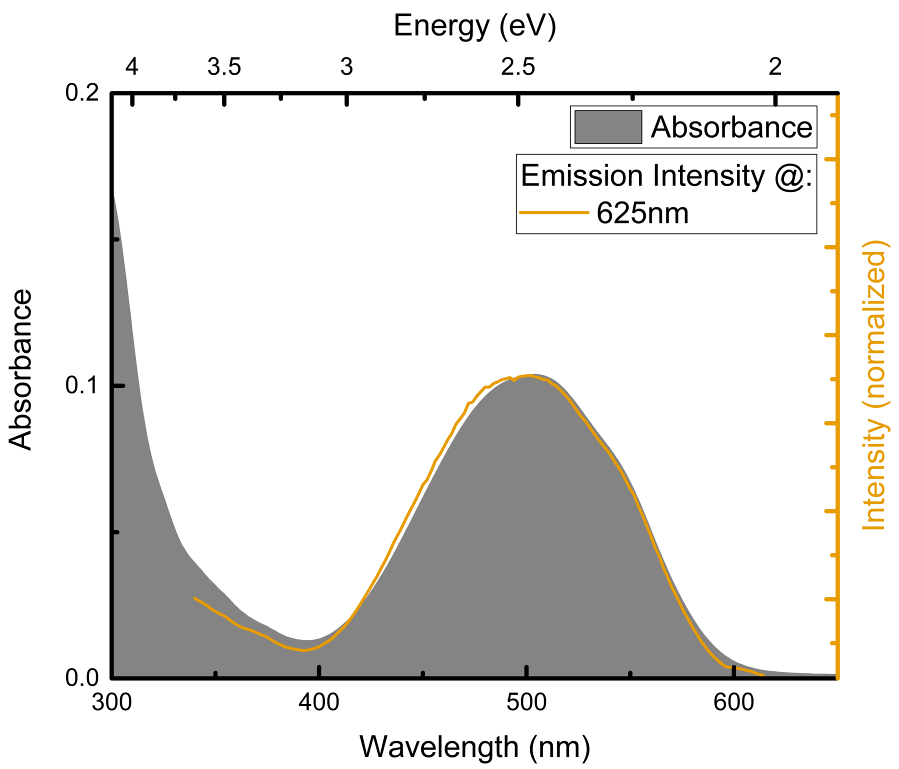

Figure S23. Absorption of [Fe(coohphtmeimb)$_2$]PF_6 in acetonitrile (grey area) and luminescence excitation spectrum of the same sample probed at 625 nm.

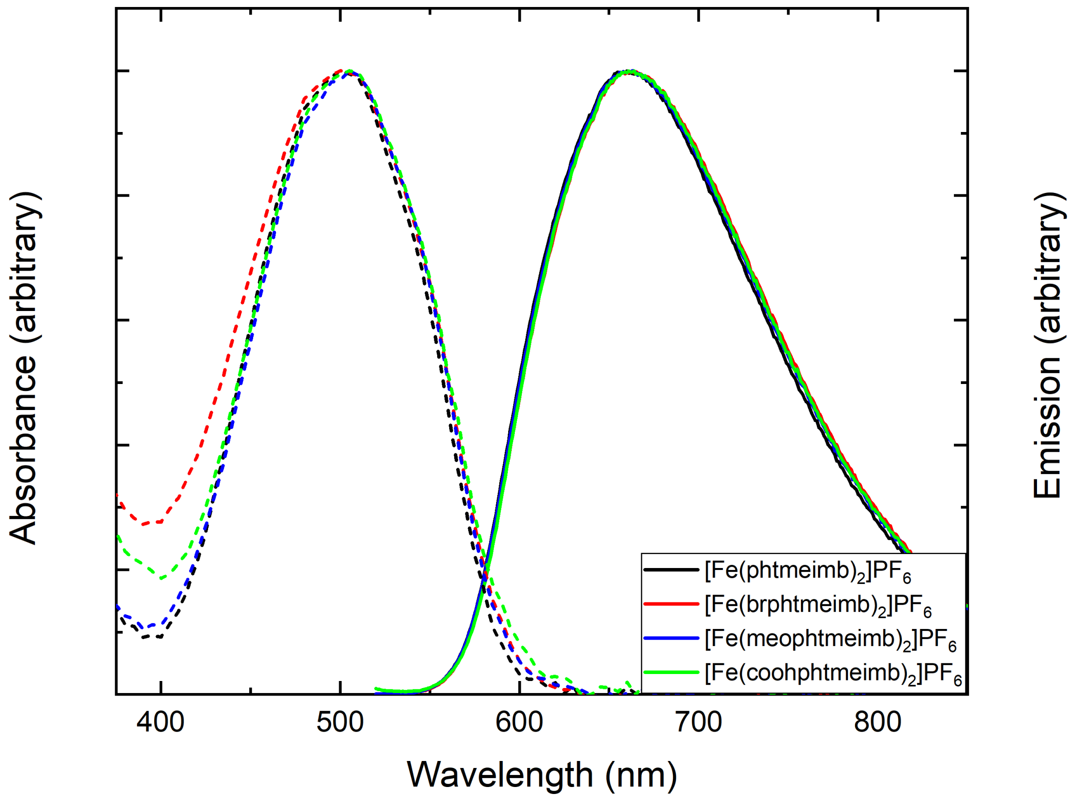

Figure S24. Normalized UV/Vis absorption spectra (dashed lines) and emission spectra (solid lines) of [Fe(phtmeimb)$_2$]PF$_6$,[3] [Fe(brphtmeimb)$_2$]PF$_6$, [Fe(meophtmeimb)$_2$]PF$_6$, and [Fe(coohphtmeimb)$_2$]PF$_6$. All complexes were measured in acetonitrile except for absorption of [Fe(coohphtmeimb)$_2$]PF$_6$ which was measured in methanol (to enable higher concentrations).

S9. Transient Absorption Spectroscopy

Transient absorption (TA) spectroscopy was performed using an in-house built setup. Basis of this setup is a Spitfire Pro XP (Spectra Physics) laser amplifier system that produces ~80 fs pulses at a central wavelength of 796 nm at 1 kHz repetition rate. The amplifier output is divided into two parts that each pump co-linear optical parametric amplifiers (TOPAS-C, Light Conversion). One of the TOPAS generates the pump beam (wavelength roughly set to the absorption maximum of each sample, ~500 nm), while the other one generates a NIR beam (1350 nm) that is focused onto a 5 mm CaF_2 crystal to generate a supercontinuum probe beam. The delay between pump and probe beams is introduced by a computer-controlled delay stage (Aerotech) placed in the probe beam's path. After supercontinuum generation the probe pulses are split into two parts: the former being focused to ~100 μm spot size and overlapping with the pump pulse in the sample volume, and the latter serving as a reference. After passing the sample the probe beam is collimated again and relayed onto the entrance slit of a prism spectrograph. The reference beam is directly relayed on said spectrograph. Both beams are then dispersed onto a double photodiode array, each holding 512 elements (Pascher Instruments). The excitation power of the pulses was set to 1 mW at ~500 nm. Mutual polarization between pump and probe beams was set to the magic angle (54.7°) by placing a Berek compensator in the pump beam. Time-resolution of the setup after dispersion correction is estimated to be ≤150 fs.

Solutions of $[Fe(brphtmeimb)_2]PF_6$ and $[Fe(meophtmeimb)_2]PF_6$ in acetonitrile (from Sigma-Aldrich Sure/Seal Bottle) was filled in 1 mm optical path length cuvettes (Hellma – Optical Special Glass) and measurements performed at room temperature. For $[Fe(coohphtmeimb)_2]PF_6$ the same was done but the solvent used was methanol (from Sigma-Aldrich Sure/Seal Bottle). The measured samples were translated after each scan to avoid photodegradation. To check for stability of each

sample steady-state absorption spectra were measured before and after TA experiments, and they were found to be the same. Before analysis the measured data were corrected for group velocity dispersion (GVD – "chirp") using in-house software KiMoPack.[5] Data were fitted by using an in-house global analysis software KiMoPack[5], model used was parallel exponential decay components.

For $[Fe(meophtmeimb)_2]PF_6$ and $[Fe(coohphtmeimb)_2]PF_6$, kinetics at selected wavelengths are shown in Figure S9.1 and S9.2 respectively. The excited state decay can be accurately described by a single exponential model and a global fit to the data results in lifetimes a bit less than 2 ns for all $[Fe(phtmeimb)_2]PF_6$ derivatives, see Table 3.

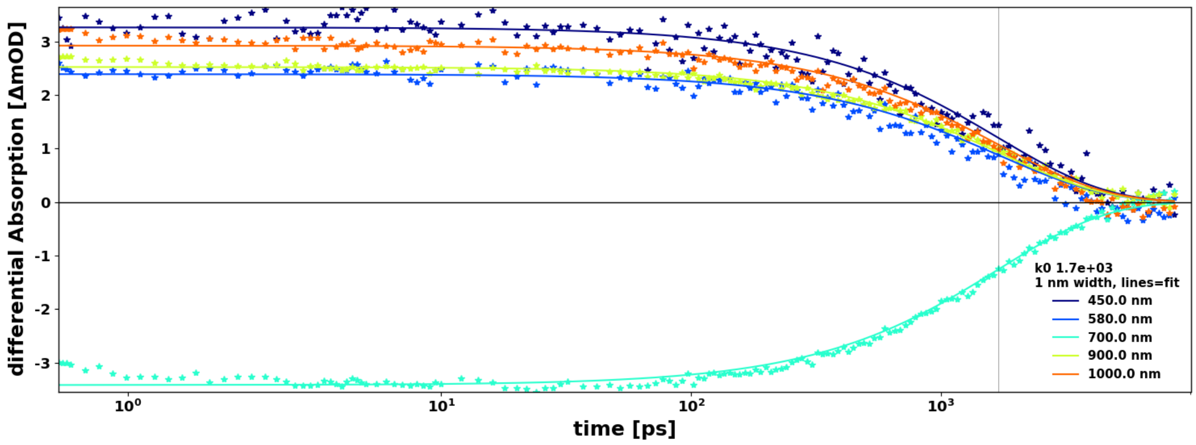

Figure S25. Kinetics at selected wavelengths of $[Fe(meophtmeimb)_2]PF_6$, also including the single exponential fit from global analysis (measured data is shown as symbols and the fit as solid lines).

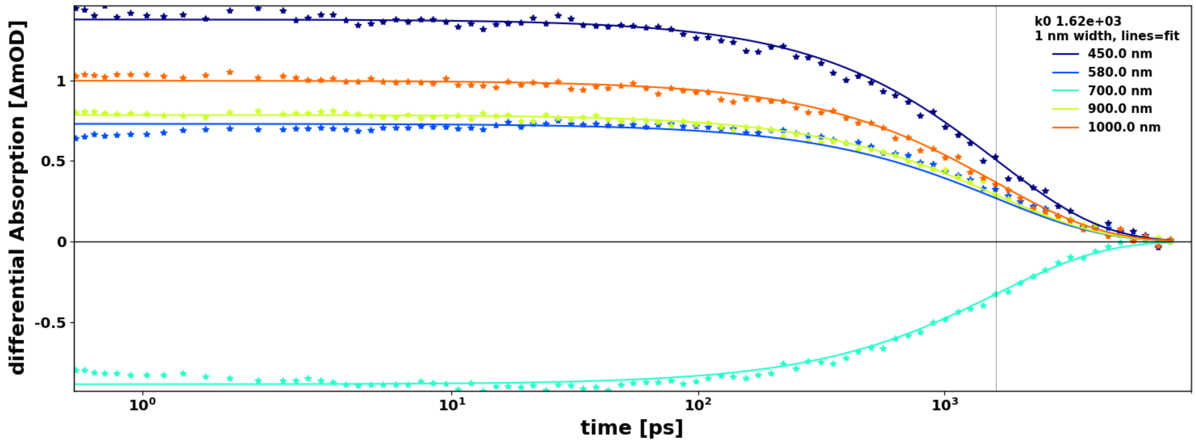

Figure S26. Kinetics at selected wavelengths of [Fe(coohphtmeimb)$_2$]PF_6, also including the single exponential fit from global analysis (measured data is shown as symbols and the fit as solid lines).

S10. Time-correlated single photon counting

Time-correlated single photon counting measurements were performed in forward mode on an Edinburgh Instruments FS5 spectrofluorometer equipped with a 475 nm diode laser (FWHM =1 ns, 20 MHz repetition rate) for excitation of dilute samples ($Abs_{475} \leq 0.05$) in a 1 x 1 cm quartz cuvette. The instrument response function of the system was determined from the light scattering of a dilute aqueous suspension of LUDOX (silica particles).

The lifetimes determined by TC-SPC are within error margins identical for the three derivatives and very close to the reported 2.0 ns of the parent complex, Figure S27. The TC-SPC lifetimes agree also reasonably well with those obtained from global fits of the transient absorption features, and the rate constants for radiative and non-radiative decay obtained with the lifetimes obtained from either method do not show significantly different trends (Table S7). The slightly shorter lifetimes found by transient absorption might be attributed to some minor degree of self-quenching in the more concentrated samples.

Table S7. Excited state lifetimes and rate constants for radiative and non-radiative decay based on TC-SPC data.

Complex	ϕ (%)	τ (ns)	k_r (10^7 s^{-1})	k_{nr} (10^8 s^{-1})
[Fe(brphtmeimb)$_2$]PF_6	1.8	1.9 (1.7*)	0.9 (1.1*)	5.2 (5.8*)
[Fe(meophtmeimb)$_2$]PF_6	1.7	1.9 (1.7*)	0.9 (1.0*)	5.2 (5.8*
[Fe(coohphtmeimb)$_2$]PF_6	1.9	1.9 (1.6*)	1.0 (1.2*)	5.2 (6.1*)

*From global fit of transient absorption data

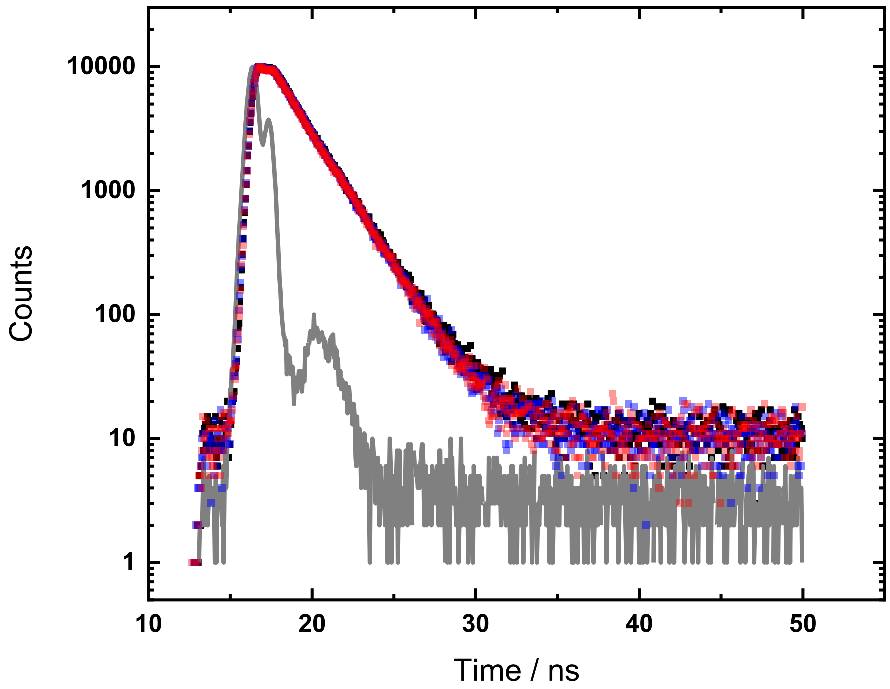

Figure S27. TC-SPC emission traces (650 nm, excitation wavelength 430 nm) of $[Fe(brphtmeimb)_2]PF_6$ (black, 1.89 ns), $[Fe(meophtmeimb)_2]PF_6$ (red, 1.86 ns) and $[Fe(coohphtmeimb)_2]PF_6$ (black, 1.86 ns) in acetonitrile solution. Indicated lifetimes from single exponential fits with numerical deconvolution with the instrument response function (grey).

S11. Quantum chemical calculations

All the quantum chemical calculations for the open-shell iron complexes $[Fe(phtmeimb)_2]^+$, $[Fe(brphtmeimb)_2]^+$, $[Fe(meophtmeimb)_2]^+$ and $[Fe(coohphtmeimb)_2]^+$ were performed with density functional theory (DFT) and B3LYP* functional together with the 6-311G(d) basis set for all included atoms.[6-9] These calculations were carried out with the Gaussian09 revision A.02 package.[10] The Ultrafine grid defined in Gaussian09 was set up in all the calculations. Solvent effects were included by adding a polarizable continuum model (PCM) with the dielectric constant of acetonitrile. Structural energy minimizations were performed for the doublet, quartet and hextet states as well as energy single point calculations in the corresponding geometries at the three different states. The relaxed geometries were validated by frequency analysis. In Tables S8-S12, the results for all studied states of the complexes are given.

Table S8. Spin density plots for the relaxed geometries in the doublet (^{2}GS), quartet (^{4}MC) and hextet (^{6}MC) multiplicities for $[Fe(phtmeimb)_2]^+$, $[Fe(brphtmeimb)_2]^+$, $[Fe(meophtmeimb)_2]^+$ and $[Fe(coohphtmeimb)_2]^+$ complexes.

	**$[Fe(phtmeimb)_2]^+$**	**$[Fe(brphtmeimb)_2]^+$**	**$[Fe(meophtmeimb)_2]^+$**	**$[Fe(coohphtmeimb)_2]^+$**
**^{2}GS**	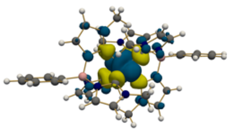	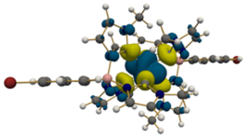	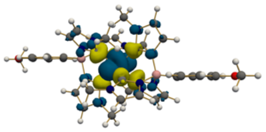	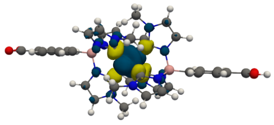
**^{4}MC**	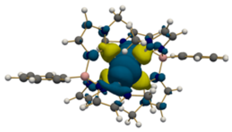	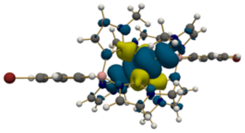	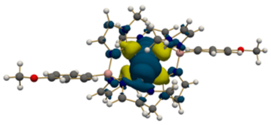	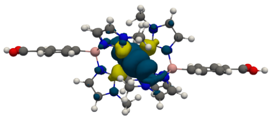
**^{6}MC**	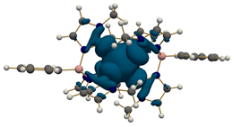	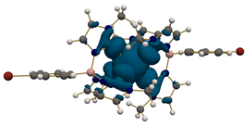	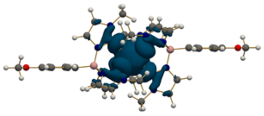	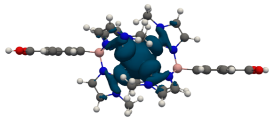

Table S9. Calculated energy and Mulliken spin density of $[Fe(phtmeimb)_2]^+$ by B3LYP*/6-311G(d). Metal-ligand average bond distances, energies and iron spin densities corresponded to the optimized structure at doublet (^{2}GS), quartet (^{4}MC) and hextet (^{6}MC) multiplicity at each of the three listed multiplicities. All energies are relative to the energy of the fully relaxed ground state structure in eV.

$[Fe(phtmeimb)_2]^+$ Relaxed Geometry	Average (M-L) Distance (Å)	^{2}GS Energy (eV)	^{4}MC Energy (eV)	^{6}MC Energy (eV)	Fe Spin Density |^{2}GS|^{4}MC|^{6}MC
^{2}GS	2.02	0.00	2.19	3.95	|1.08|2.69|4.05
^{4}MC	2.15	0.76	1.36	2.92	|1.16|2.84|4.12
^{6}MC	2.23	1.21	1.88	2.28	|1.21|2.93|4.05

Table S10. Calculated energy and Mulliken spin density of $[Fe(brphtmeimb)_2]^+$ by B3LYP*/6-311G(d). Metal-ligand average bond distances, energies and iron spin densities corresponded to the optimized structure at doublet (^{2}GS), quartet (^{4}MC) and hextet (^{6}MC) multiplicity at each of the three listed multiplicities. All energies are relative to the energy of the fully relaxed ground state structure in eV.

$[Fe(brphtmeimb)_2]^+$ Relaxed Geometry	Average (M-L) Distance (Å)	^{2}GS Energy (eV)	^{4}MC Energy (eV)	^{6}MC Energy (eV)	Fe Spin Density |^{2}GS|^{4}MC|^{6}MC
^{2}GS	2.02	0.00	2.19	3.95	|1.082.69|4.05
^{4}MC	2.15	0.76	1.35	2.91	|1.16|2.84|4.12
^{6}MC	2.23	1.21	1.87	2.28	|1.21|2.93|4.18

Table S11. Calculated energy and Mulliken spin density of $[Fe(meophtmeimb)_2]^+$ by B3LYP*/6-311G(d). Metal-ligand average bond distances, energies and iron spin densities corresponded to the optimized structure at doublet (^{2}GS), quartet (^{4}MC) and hextet (^{6}MC) multiplicity at each of the three listed multiplicities. All energies are relative to the energy of the fully relaxed ground state structure in eV.

$[Fe(meophtmeimb)_2]^+$ Relaxed Geometry	Average (M-L) Distance (Å)	^{2}GS Energy (eV)	^{4}MC Energy (eV)	^{6}MC Energy (eV)	Fe Spin Density |^{2}GS|^{4}MC|^{6}MC
^{2}GS	2.02	0.00	2.19	3.94	|1.08|2.69|4.05
^{4}MC	2.15	0.76	1.36	2.91	|1.16|2.84|4.12
^{6}MC	2.23	1.21	1.88	2.28	|1.21|2.94|4.18

Table S12. Calculated energy and Mulliken spin density of $[Fe(coohphtmeimb)_2]^+$ by B3LYP*/6-311G(d). Metal-ligand average bond distances, energies and iron spin densities corresponded to the optimized structure at doublet (^{2}GS), quartet (^{4}MC) and hextet (^{6}MC) multiplicity at each of the three listed multiplicities. All energies are relative to the energy of the fully relaxed ground state structure in eV.

$[Fe(coohphtmeimb)_2]^+$ Relaxed Geometry	Average (M-L) Distance (Å)	^{2}GS Energy (eV)	^{4}MC Energy (eV)	^{6}MC Energy (eV)	Fe Spin Density |^{2}GS|^{4}MC|^{6}MC
^{2}GS	2.02	0.00	2.19	3.95	|1.08|2.69|4.05
^{4}MC	2.15	0.76	1.36	2.91	|1.16|2.84|4.12
^{6}MC	2.23	1.21	1.88	2.28	|1.21|2.93|4.18

S12. References

1. Sheldrick, G. M. *A short history of SHELX. Acta Crystallogr. A,* **2008**, *64*, 112–122.

2. Dolomanov, O. V.; Bourhis, L. J.; Gildea, R. J.; Howard, J. A. K.; Puschmann, H. OLEX2: a complete structure solution, refinement and analysis program. *J. Appl. Cryst.* **2009**, *42*, 339–341.

3. Kjær, K. S.; Kaul, N.; Prakash, O.; Chábera, P.; Rosemann, N. W.; Honarfar, A.; Gordivska, O.; Fredin, L. A.; Bergquist, K.; Häggström, L.; Ericsson, T.; Lindh, L.; Yartsev, A.; Styring, S.; Huang, P.; Uhlig, J.; Bendix, J.; Strand, D.; Sundström, V.; Persson, P.; Lomoth, R.; Wärnmark, K. Luminescence and reactivity of a charge-transfer excited iron complex with nanosecond lifetime. *Science* **2019**, *363*, 249–253.

4. Chábera, P.; Liu, Y.; Prakash, O.; Thyrhaug, E.; Nahhas, A.; Honarfar, A.; Essén, S.; Fredin, L.A.; Harlang, T.C.B.; Kjær, K.S.; Handrup, K.; Ericson, F.; Tatsuno, H.; Morgan, K.; Schnadt, J.; Häggström, L.; Ericsson, T; Sobkowiak, A.; Lidin, S.; Huang, P.; Styring, S.; Uhlig, J.; Bendix, J.; Lomoth, R.; Sundström, V.; Persson, P.; Wärnmark, K. A low-spin Fe(iii) complex with 100-ps ligand-to-metal charge transfer photoluminescence. *Nature* **2017**, *543*, 695–699.

5. Muller, C.; Pascher, T.; Eriksson, A.; Chabera, P.; Uhlig, J. KiMoPack: A python Package for Kinetic Modeling of the Chemical Mechanism *J. Phys. Chem. J. Phys. Chem. A* **2022**, *126*, 25, 4087–4099.

6. Becke, A. D. A Density functional thermochemistry III. The role of exact exchange. *J. Chem. Phys.* **1993**, *98*, 7, 5648–5652.

7. Reiher, M.; Salomon, O.; Artur Hess, B. Reparameterization of hybrid functionals based on energy differences of states of different multiplicity. *Theor. Chem. Acc.* **2001**, *107*, 1, 48–55.

8. Krishnan, R.; Binkley, J. S.; Seeger, R.; Pople, J. A. Self-consistent molecular orbital methods XX. A basis set for correlated wave functions. *J. Chem. Phys.* **1980**, *72*, 1, 650–654.

9. McLean, A. D.; Chandler, G. S. Contracted Gaussian basis sets for molecular calculations. I. Second row atoms, Z=11-18. *J. Chem. Phys.* **1980**, *72*, 10, 5639–5648.

10. Frisch, M. J.; Trucks, G. W.; Schlegel, H. B.; Scuseria, G. E.; Robb, M. A.; Cheeseman, J. R.; Scalmani, G.; Barone, V.; Mennucci, B.; Petersson, G. A.; Nakatsuji, H.; Caricato, M.; Li, X.; Hratchian, H. P.; Izmaylov, A. F.; Bloino, J.; Zheng, G.; Sonnenberg, J. L.; Hada, M.; Ehara, M.; Toyota, K.; Fukuda, R.; Hasegawa, J.; Ishida, M.; Nakajima, T.; Honda, Y.; Kitao, O.; Nakai, H.; Vreven, T.; Montgomery, J. A. J.; Peralta, J. E.; Ogliaro, F.; Bearpark, M.; Heyd, J. J.; Brothers, E.; Kudin, K. N.; Staroverov, V. N.; Kobayashi, R.; Normand, J.; Raghavachari, K.; Rendell, A.; Burant, J. C.; Iyengar, S. S.; Tomasi, J.; Cossi, M.; Rega, N.; Millam, J. M.; Klene, M.; Knox, J. E.; Cross, J. B.; Bakken, V.; Adamo, C.; Jaramillo, J.; Gomperts, R.; Stratmann, R. E.; Yazyev, O.; Austin, A. J.; Cammi, R.; Pomelli, C.; Ochterski, J. W.; Martin, R. L.; Morokuma, K.; Zakrzewski, V. G.; Voth, G. A.; Salvador, P.; Dannenberg, J. J.; Dapprich, S.; Daniels, A. D.; Farkas, O.; Foresman, J. B.; Ortiz, J. V.; Cioslowski, J.; Fox, D. J. *Gaussian 09, Revision A.02. Gaussian, Inc.: Wallingford CT* **2009**.
